# Fragile X Mental Retardation Protein Regulates Proliferation and Differentiation of Adult Neural Stem/Progenitor Cells

**DOI:** 10.1371/journal.pgen.1000898

**Published:** 2010-04-08

**Authors:** Yuping Luo, Ge Shan, Weixiang Guo, Richard D. Smrt, Eric B. Johnson, Xuekun Li, Rebecca L. Pfeiffer, Keith E. Szulwach, Ranhui Duan, Basam Z. Barkho, Wendi Li, Changmei Liu, Peng Jin, Xinyu Zhao

**Affiliations:** 1 Department of Neurosciences, University of New Mexico School of Medicine, Albuquerque, New Mexico, United States of America; 2 Department of Human Genetics, Emory University School of Medicine, Atlanta, Georgia, United States of America; University of Minnesota, United States of America

## Abstract

Fragile X syndrome (FXS), the most common form of inherited mental retardation, is caused by the loss of functional fragile X mental retardation protein (FMRP). FMRP is an RNA–binding protein that can regulate the translation of specific mRNAs. Adult neurogenesis, a process considered important for neuroplasticity and memory, is regulated at multiple molecular levels. In this study, we investigated whether Fmrp deficiency affects adult neurogenesis. We show that in a mouse model of fragile X syndrome, adult neurogenesis is indeed altered. The loss of Fmrp increases the proliferation and alters the fate specification of adult neural progenitor/stem cells (aNPCs). We demonstrate that Fmrp regulates the protein expression of several components critical for aNPC function, including CDK4 and GSK3β. Dysregulation of GSK3β led to reduced Wnt signaling pathway activity, which altered the expression of neurogenin1 and the fate specification of aNPCs. These data unveil a novel regulatory role for Fmrp and translational regulation in adult neurogenesis.

## Author Summary

Fragile X syndrome, the most common cause of inherited mental retardation, results from the loss of functional Fragile X mental retardation protein (FMRP). FMRP is an RNA–binding protein and is known to bind to specific mRNAs and to regulate their translation both *in vitro* and *in vivo*. Adult neurogenesis, a process considered important for neuroplasticity and memory, is regulated at multiple molecular levels. Here we show that Fmrp could regulate the proliferation and fate specification of adult neural progenitor/stem cells (aNPCs). These data unveil a novel regulatory role for Fmrp in adult neurogenesis.

## Introduction

Fragile X syndrome, one of the most common forms of inherited mental retardation, is caused by the functional loss of fragile X mental retardation protein (FMRP/Fmrp) [1]. Patients with fragile X syndrome show an array of deficits in motor control, cognition, learning, and memory, although their overall brain morphology is generally normal. Fmrp is a selective RNA-binding protein that forms a messenger ribonucleoprotein (mRNP) complex that can associate with polyribosomes. Evidence suggests that Fmrp is involved in the post-transcriptional regulation of protein synthesis [2]–[4]. Studies from both human patient brain tissues and Fmrp mutant mice suggest that Fmrp is involved in synaptic plasticity and dendritic development. Fmrp mutant mice are found to perform poorly in highly challenging learning tests [5], particularly the hippocampus-dependent trace learning test [6],[7], suggesting that Fmrp is necessary especially for complex learning that requires an intact hippocampus. However, how the functional deficiency of Fmrp results in learning and memory deficits remains unclear.

Neurogenesis persists throughout life in two germinal zones, the subgranular zone (SGZ) in the dentate gyrus (DG) of the hippocampus and the subventricular zone (SVZ) of the lateral ventricles. The neurons produced in the DG during adulthood are known to integrate into the existing circuitry of the hippocampus, and young neurons show greater synaptic plasticity than mature neurons under identical conditions [8],[9]. Although the specific purpose of adult neurogenesis is still being debated, mounting evidence points to an important role in adult neuroplasticity [9]–[11]. It has been suggested that new neurons in the DG are critical for hippocampus-dependent learning [10],[12],[13]. Indeed, blocking of adult neurogenesis using generic anti-proliferative drugs or radiation can lead to deficits in learning and memory [14]–[16]. More recent direct evidence has come from inducing the death of new neurons in the hippocampus [17]–[19] and from inhibiting the Wnt signaling pathway in the hippocampus using retrovirus [20]. Adult neurogenesis is regulated at many levels by both extrinsic factors, such as physiological and pathological conditions, and intrinsic factors, such as genetic and epigenetic programs [21]. Although both adult hippocampal neurogenesis and learning are altered in several pathological conditions, such as stress, diabetes, neurological diseases, strokes, and traumatic injuries, the link between adult neurogenesis and mental retardation, a deficiency in learning and memory, remains elusive [9]–[11].

The cellular basis of adult neurogenesis is adult neural progenitor/stem cells (aNPCs). The maintenance and differentiation of aNPCs are tightly controlled by intricate molecular networks [22]. Despite exhaustive efforts devoted to understanding transcriptional regulation in adult neurogenesis, the role of translational control by RNA-binding proteins, such as Fmrp, has gone largely unexplored. Recently, Fmrp was found to be required for the maintenance of *Drosophila* germline stem cells [23]; however, its function in mammalian embryonic neurogenesis is controversial [24],[25]. Whether and how Fmrp regulates neural stem cells in the adult mammalian brain and the implications for learning and memory have not been established.

Here we show that loss of Fmrp *in vitro* and *in vivo* led to altered adult neurogenesis and impaired learning. Fmrp-deficient aNPCs displayed increased proliferation and decreased neuronal differentiation, but increased glial differentiation. We identified specific mRNAs regulated by Fmrp in stem cell proliferation and differentiation, including glycogen synthase kinase 3β (GSK3ß), a negative regulator of ß-catenin and the canonical Wnt signaling pathway that has been implicated in adult neurogenesis [26],[27]. The loss of Fmrp resulted in reduced ß-catenin levels and a defective Wnt signaling pathway, which in turn led to the downregulation of neurogenin1 (Neurog1), which is an early initiator of neuronal differentiation and an inhibitor of astrocyte differentiation [28],[29]. These data not only reveal a novel regulatory role for Fmrp in adult neurogenesis, but also provide direct evidence that adult neurogenesis could be a factor in the pathogenesis of fragile X mental retardation.

## Results

### Loss of Fmrp alters the proliferation and fate specification of aNPCs

**Figure 1:**
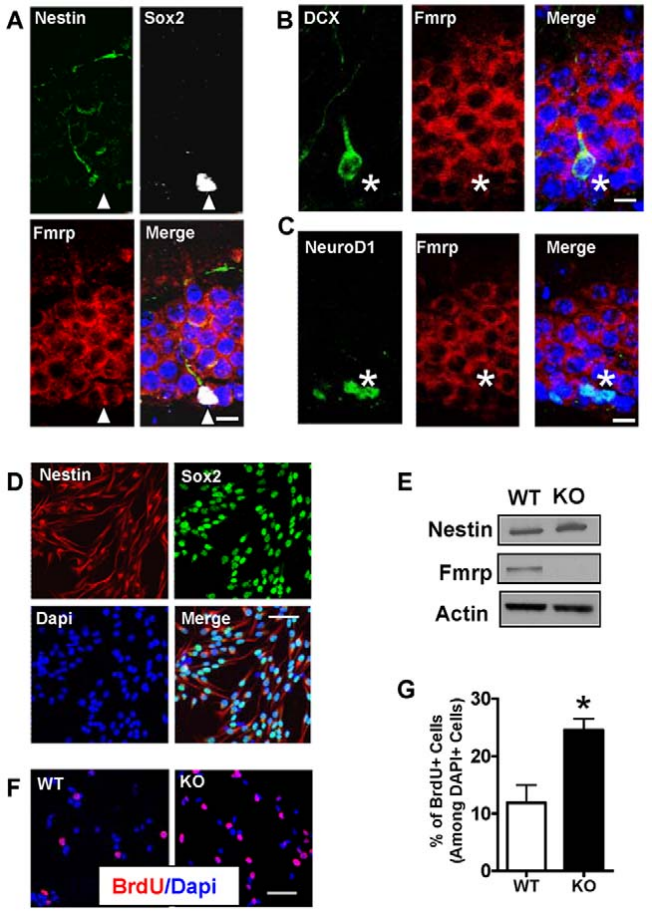
Fmrp is expressed in aNPCs and new neurons in the adult DG, and the loss of Fmrp leads to increased aNPC proliferation. (A) Fmrp is expressed in Sox2 (white) and Nestin (green) double-positive NPCs (arrowheads) in the granule neurons of the adult hippocampus. Arrowhead points to a positive cell located at the subgranular zone adjacent to the hilar region. (B,C) Fmrp is expressed in doublecortin (DCX)-positive (B, green) and NeuroD1-postive (C, green) newly generated neurons. Asterisks identify positive cells located at the subgranular zone adjacent to the hilar region. (A–C, Fmrp, red; Dapi, blue; Scale bars = 10 µm;). (D) aNPCs cultured under proliferating conditions expressed the neural progenitor markers Nestin (cytoplasmic, red) and Sox2 (nuclear, green; Dapi in blue). (E) Proliferating WT aNPCs, but not *Fmr1* KO aNPCs, expressed Fmrp. (F) Both WT and KO aNPCs incorporate the thymidine analog, BrdU, under proliferating conditions (BrdU, red; Dapi, blue; (D,F), Scale bars = 50 µm). (G) Quantitative analysis showing that a higher percentage of *Fmr1* KO aNPCs incorporated BrdU. (*, p<0.05; n = 3; Student's t-test; mean ± SEM).

To investigate the role of Fmrp in adult neurogenesis, we determined the expression pattern of Fmrp in the dentate gyrus (DG) of the adult hippocampus using cell type-specific markers. Consistent with published literature [30],[31], Fmrp was enriched in a majority of the granule neurons in the DG (Figure S1A), but was undetectable in either GFAP-positive or S100β-positive astrocytes (Figure S1B and S1C). Using markers specific to immature neural progenitors (NPCs) and young neurons, we discovered that Fmrp was also expressed in Sox2 and Nestin double-positive NPCs (Figure 1A), as well as in either NeuroD1-postive or doublecortin (DCX)-positive newly generated neurons (Figure 1B and 1C). The presence of Fmrp in these immature cells supports a potential function of this protein in adult neurogenesis.

To determine the functions of Fmrp in aNPCs, we isolated aNPCs from both the forebrain and the dentate gyrus (DG) of adult *Fmr1* knockout (KO) mice and wild-type (WT) controls. Due to the difficulty of obtaining large numbers of the DG aNPCs, we performed all functional assays first using forebrain aNPCs, and then confirmed our findings using the DG aNPCs. As shown below, we found that both the forebrain aNPCs and the DG aNPCs yielded similar results. Nearly all cultured aNPCs were positive for the progenitor markers Nestin and Sox2 (Figure 1D), suggesting a relative homogeneity of these primary aNPCs. Fmrp was expressed in WT aNPCs, but not in *Fmr1* KO aNPCs (Figure 1E). We pulsed the cells with BrdU for eight hours to assess the proliferation of these aNPCs (Figure 1F) and found that *Fmr1* KO aNPCs exhibited twice as much BrdU incorporation as WT aNPCs (Figure 1G). We further analyzed the cell cycle profiles of aNPCs and found that more *Fmr1* KO cells were in mitotic (G2/M) phase compared with WT controls (Figure S2, 11% higher; n = 3, p<0.02). Hence a lack of functional Fmrp led to a rise in the proliferative capability of aNPCs.

**Figure 2:**
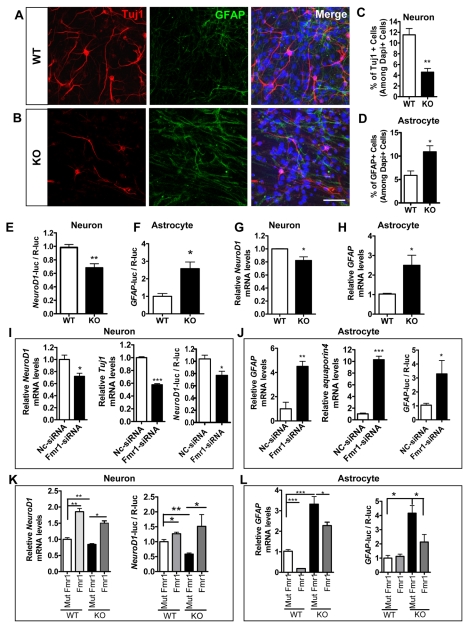
Loss of Fmrp leads to decreased neuronal differentiation but increased astrocyte differentiation. (A,B) Sample immunostained cells using cell lineage markers for quantitative cell fate determination shown in (C,D). Both WT (A) and *Fmr1* KO (B) aNPCs could differentiate into Tuj1^+^ (red) neurons and GFAP^+^ (green) astrocytes. (Scale bar = 50 µm; DAPI, nuclear staining, blue). (C,D) Quantitative analyses of differentiated aNPCs demonstrate that *Fmr1* KO aNPCs differentiated into fewer Tuj1+ neurons (C, n = 4; p<0.01) but more GFAP+ astrocytes (D, n = 6, p<0.05). Quantification was performed using an unbiased stereology method. (E,F), Luciferase reporter assay showing that differentiating *Fmr1* KO aNPCs had decreased *NeuroD1* (E; n = 4, p<0.01), but increased GFAP (F, n = 3, p<0.05) promoter activities compared with WT aNPCs. A co-transfected Renilla luciferase (R-Luc) plasmid was used as a transfection control. (G,H), Real-time PCR assays showing that *Fmr1* KO aNPCs had decreased *NeuroD1* mRNA levels (G; n = 3, p<0.05), but increased GFAP mRNA levels (H, n = 3, p<0.05) upon differentiation. The relative mRNA levels were in comparison with GAPDH mRNA. (I,J), Acute knockdown of Fmrp expression in WT aNPCs using siRNA led to decreased neuronal differentiation (I; left *NeuroD1*; middle, *Tuj1*; right, *NeuroD1*-promoter), but increased astrocyte differentiation (J; left *GFAP*; middle, *aquaporin4*; right, *GFAP*-promoter); (K,L), Exogenously expressed WT Fmrp, but not mutant (I304N) Fmrp, could enhance neuronal differentiation (K) and repress astrocyte differentiation (L) in *Fmr1* KO aNPCs. GAPDH mRNA levels were used as internal controls for real-time PCR analyses. Data are presented as mean ± SEM; *, p<0.05, **, p<0.01, ***, p<0.01, Student's t-test.

To assess the effect of Fmrp on aNPC differentiation, both WT and *Fmr1* KO forebrain aNPCs were differentiated for three days, and the phenotypes of differentiated cells were determined using several independent assays. First, differentiated cells were stained using cell lineage-specific antibodies, β-III tubulin (Tuj1) for neurons and glial fibrillary acidic protein (GFAP) for astroglia [32],[33]. Both WT and *Fmr1* KO aNPCs could be induced to differentiate into neurons and astrocytes (Figure 2A and 2B); however, *Fmr1* KO aNPCs exhibited a 60.4% decrease in neuronal differentiation (Figure 2C) and a 74.9% increase in astrocyte differentiation (Figure 2D) compared with WT aNPCs. Under our culture conditions, only differentiated astrocytes, not proliferating aNPCs, expressed GFAP (data not shown). To validate our immunocytochemical data, we then assessed the neuronal differentiation of aNPCs by measuring the promoter activity of a pan-neuronal transcription factor, neurogenic differentiation 1 (NeuroD1), and astrocyte differentiation by measuring the promoter activity of *GFAP* using two well-characterized promoter constructs [34]–[37]. We found that in *Fmr1* KO aNPCs, *NeuroD1* promoter activity decreased by 31.4% (Figure 2E), whereas *GFAP* promoter activity increased by 73.4% (Figure 2F), which is consistent with our immunocytochemistry results. Finally, using real-time quantitative PCR, we further demonstrated that differentiating *Fmr1* KO aNPCs had 17.8% reduced *NeuroD1* mRNA (Figure 2G, n = 3, p<0.05), but 1.5×-fold increased *GFAP* mRNA (Figure 2H; n = 3, p<0.05) levels. Since the above three methods, immunostaining, promoter activity assay, and real-time PCR, yielded consistent results, we used these assays as interchangeable methods for assessing aNPC differentiation in subsequent experiments. The increased proportion of astrocytes in differentiating *Fmr1* KO aNPCs was not due to an increased proliferation of newly differentiated astrocytes, because GFAP^+^ astrocytes differentiated from *Fmr1* KO aNPCs did not incorporate more BrdU compared with those from WT aNPCs (data not shown). The differentiation to oligodendrocytes was no different between *Fmr1* KO and WT aNPCs (data not shown).

To confirm that the altered fate specification of *Fmr1* KO aNPCs was due to the loss of functional Fmrp, we used siRNA (*Fmr1*-siRNA, Figure S3) to knock down Fmrp expression in WT aNPCs. We found that acute knockdown of Fmrp expression in WT aNPCs led to both reduced *NeuroD1* (Figure 2I, left, n = 4, p<0.05) and *Tuj1* (Figure 2I, middle, n = 4, p<0.001) mRNA levels, as well as diminished *NeuroD1* promoter activity (Figure 2I, right, n = 6, p<0.05) compared with aNPCs transfected with a nonsilencing control siRNA (NC-siRNA). On the other hand, acute knockdown of Fmrp resulted in increased mRNA levels of both *GFAP* (Figure 2J, left; n = 4 p<0.01) and another astrocyte marker *aquaporin4* [38],[39] (Figure 2J, middle, n = 4, p<0.001), as well as enhanced *GFAP* promoter activity (Figure 2J, right, n = 6, p<0.05). Furthermore, exogenously expressed WT Fmrp, but not mutant (I304N) Fmrp, which is unable to bind polyribosomes [40], rescued both the neuronal (Figure 2K) and the astrocyte (Figure 2L) differentiation deficits associated with *Fmr1* KO cells.

We then confirmed that aNPCs isolated from *Fmr1* KO DG had similar reductions in neuronal differentiation and increases in astrocyte differentiation (Figure S4A, S4B, S4C, S4D) as *Fmr1* KO aNPCs derived from forebrain. In addition, acute knockdown of Fmrp in the WT DG aNPCs resulted in phenotypes in neuronal and astrocyte differentiation (Figure S4E, S4F, S4G, S4H) similar to those we observed in forebrain aNPCs. Together, these results suggest that the loss of Fmrp alters both the proliferation and fate specification of aNPCs.

### Loss of Fmrp alters adult neurogenesis *in vivo*

**Figure 3:**
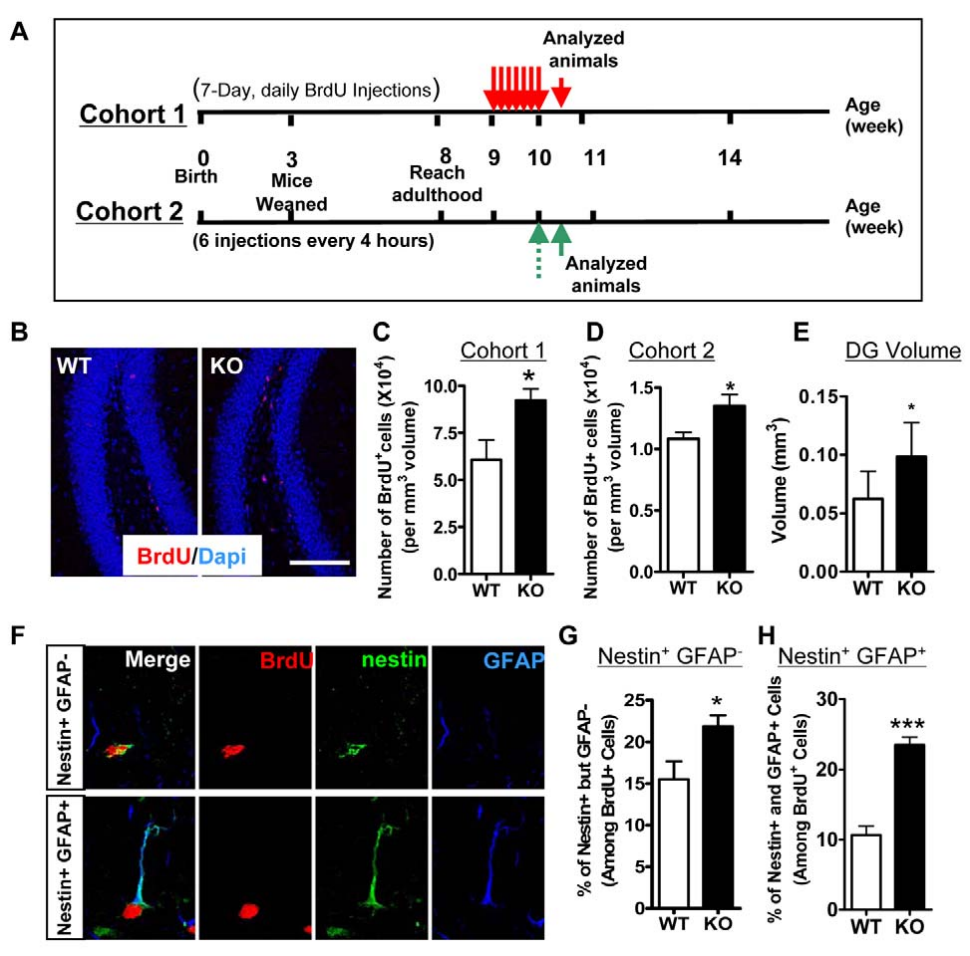
Loss of Fmrp alters the proliferation of neural stem and progenitor cells *in vivo*. (A) Experimental scheme for assessing cell proliferation in the adult hippocampus. Cohort 1 animals had the same injection paradigm as Figure 4 and were therefore used to assess new cell survival. Cohort 2 animals were used to evaluate cell proliferation in the DG. (B) Examples of WT and *Fmr1* KO brain sections stained with an antibody against BrdU (red) and DAPI (blue) for *in vivo* neurogenesis analyses (scale bar = 100 µm). (C) The dentate gyrus (DG) of *Fmr1* KO mice exhibited increased BrdU^+^ cells analyzed at one day after a 7-day regimen of daily BrdU injections, suggesting increased proliferation (Cohort 1: n = 3 WT; n = 4 KO). (D) At 4 hours post-BrdU injection (6 injections within 24 hours), the number of BrdU+ cells normalized to volume of the DG was also higher in *Fmr1* KO mice (p<0.05). (D–I, Cohort 2: n = 7 WT; n = 6 KO). (E) *Fmr1* KO mice also had increased DG volume (size) (p<0.05). (F) Single intensity projection confocal z-series showing two different types of BrdU+ cells in the DG of the hippocampus. Upper panel, BrdU+ (red), Nestin+ (green), and GFAP− (blue) progenitor cells; Lower panel, BrdU+ (red), Nestin+ (green), and GFAP+ (blue) stem-like cells. (G) The DG of *Fmr1* KO mice exhibited increased proliferation of progenitor (BrdU^+^ Nestin^+^, GFAP^−^) cells analyzed at 4 hours following 6 BrdU injections within a 24-hour period. (H) The DG of *Fmr1* KO mice exhibited increased proliferation of stem (BrdU^+^ Nestin^+^, GFAP^+^) cells analyzed at 4 hours after 6 BrdU injections within a 24-hour period. (n = 7 WT; n = 6 KO). Data are presented as mean ± SEM; *, p<0.05, **, p<0.01, ***, p<0.01, Student's t-test.

**Figure 4:**
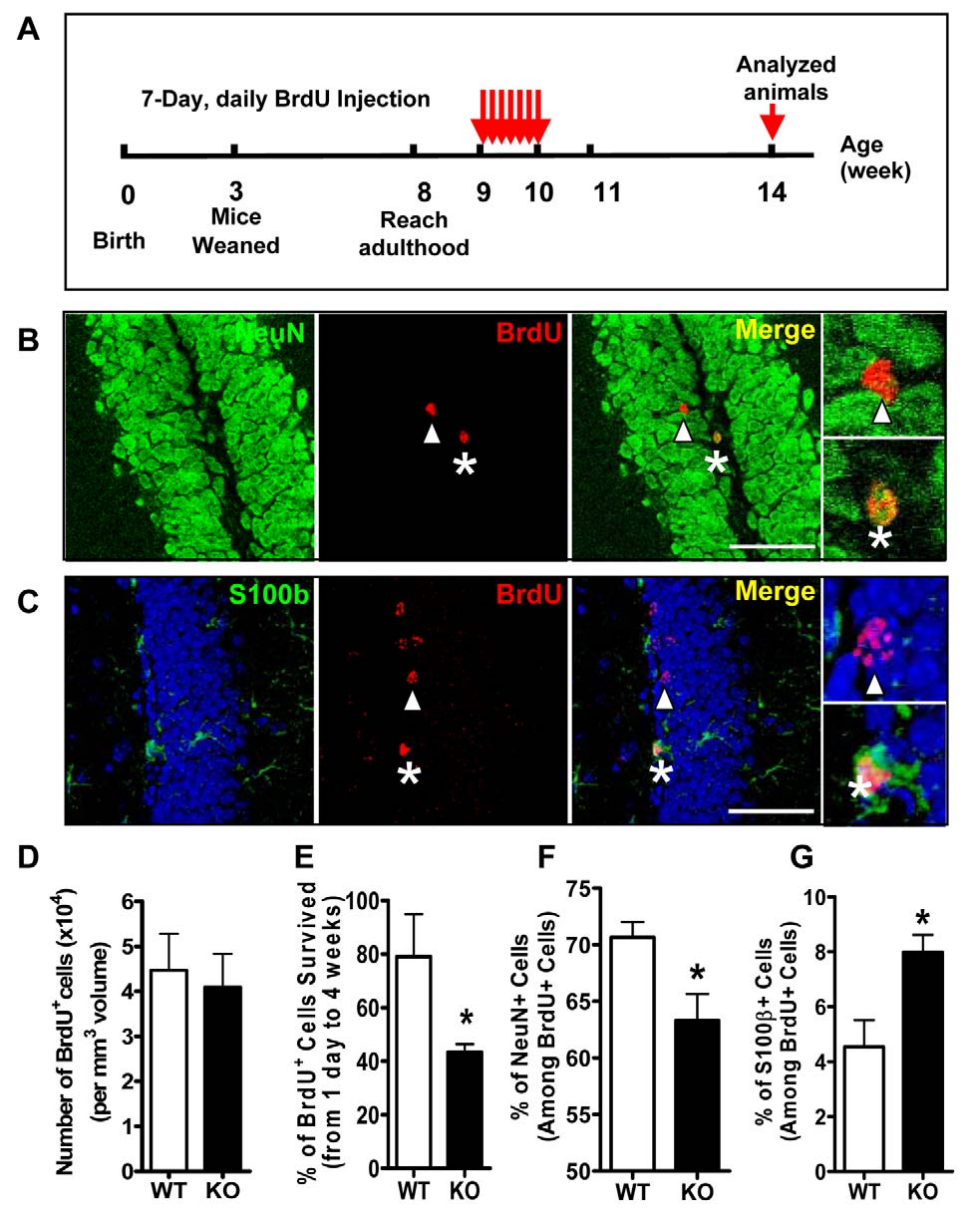
Loss of Fmrp alters the differentiation of neural stem and progenitor cells *in vivo*. (A) Experimental scheme for assessing new cell survival and differentiation in the adult hippocampus. (B,C) Sample confocal images showing newborn cells that had differentiated into NeuN^+^ neurons (B, asterisk) or S100β^+^ astrocytes (C, asterisk). Asterisks, but not arrowheads, indicate BrdU^+^ cells that have differentiated into either a neuron (H) or an astrocyte (E). (D) At 4 weeks post-labeling, BrdU^+^ cells in the *Fmr1* KO DG were no different from WT mice (n = 7 WT; n = 9 KO). (E) At 4 weeks post-BrdU injection, brains were analyzed for survival of newborn cells in the DG. The ratio of BrdU^+^ cells at 4 weeks post-injection (numbers used for D) over 1 day post-injection (average of BrdU+ cells shown in Figure 3D) indicated that *Fmr1* KO mice had fewer surviving newborn cells in the DG. (F,G) Quantitative analysis indicated that newborn cells in *Fmr1* KO mice differentiated into a lower percentage of neurons (F) but a higher percentage of astrocytes (G) compared with WT mice (n = 9 WT; n = 9 KO). All data are shown as mean ± SEM, and Student's t-test was used for all the analyses. *, p<0.05, **, p<0.01 ***, p<0.001.

To investigate the role of Fmrp in adult neurogenesis *in vivo*, we assessed the proliferation, survival, and differentiation of endogenous aNPCs in both WT and *Fmr1* KO mice. Newborn cells were distinguished by the incorporation of BrdU administered through intraperitoneal injections into adult mice using two cohorts of mice (Figure 3A). Cohort 1 animals (Figure 3C) had the same injection paradigm as those mice used for the differentiation assay (Figure 4); therefore, they were used to assess new cell survival. Cohort 2 animals were used to evaluate cell proliferation in the DG. Quantitative histological analysis at one day following a seven-day regimen of daily BrdU injection (Cohort 1) showed that *Fmr1* KO mice had 52.0% more BrdU-positive cells compared with WT mice (Figure 3C). To further assess the proliferation of aNPCs without the confound of cell survival in *Fmr1* KO mice, we gave mice six doses of BrdU injection within 24 hours to label the entire proliferating population in the DG based on a published paradigm [41] and analyzed the mice at four hours after the last BrdU injection (Figure 3D, Cohort 2). We found that *Fmr1* KO mice had 53.2% more BrdU-positive cells compared with WT mice (Figure 3D, p<0.001). Since the volume of the DG is also increased in *Fmr1* KO mice (Figure 3E, p<0.05) and the above data were normalized to the DG volume, the total number of BrdU-positive cells was even higher in KO mice compared with WT controls. It has been shown that the adult DG contains at least two types of proliferating immature cells that can be labeled by BrdU: one type is GFAP+ and Nestin+ (Figure 3F lower panel) and might be stem cells, whereas the other type is GFAP− and Nestin+ (Figure 3F upper panel) and more likely to be progenitor cells [10],[42]. To determine which types of cells exhibited increased BrdU incorporation in *Fmr1* KO mice, we stained the brain sections with antibodies against BrdU, GFAP, and Nestin (Figure 3F). We found that the *Fmr1* KO DG had increased BrdU incorporation in both the Nestin+/GFAP− cell population (Figure 3G, 40.8% increase, p<0.05) and the Nestin+/GFAP+ cell population (Figure 3H, p<0.001, 1.2-fold increase). The proliferation of astrocytes (BrdU+, GFAP+, Nestin− cells) was no different between WT and *Fmr1* KO mice (data not shown). Cell proliferation in the SVZ was also 1.1-fold higher in *Fmr1* KO mice (p<0.05). Thus Fmrp deficiency may lead to increased proliferation of both stem and progenitor cells.

The long-term survival and differentiation of BrdU-labeled cells was evaluated by analyzing the labeled cells at four weeks after BrdU injections (Figure 4A–4C). The number of BrdU^+^ cells at four weeks post-injection was no different between WT and *Fmr1* KO mice (Figure 4D); therefore, the percentage of BrdU^+^ cells that survived from one day to four weeks post-BrdU administration is significantly lower in *Fmr1* KO mice compared with WT mice (Figure 4E, p<0.05). Hence Fmrp deficiency may also lead to reduced survival of young neurons.

Since we observed altered neuronal and astrocyte differentiation of *Fmr1* KO aNPCs *in vitro* (Figure 2), we then used triple fluorescence immunostaining with antibodies for mature neurons (NeuN) and astrocytes (S100β) to further determine the fate of differentiated aNPCs *in vivo* (Figure 4B and 4C). Consistent with our *in vitro* observation, we found that in *Fmr1* KO mice, the percentage of BrdU^+^ cells that are NeuN^+^ neurons was 10.4% lower (Figure 3F, p<0.05), whereas the percentage of BrdU-positive cells that are S100β^+^ astrocytes was 75.7% higher compared with WT mice (Figure 4G, p<0.05). In addition, the expression levels of NeuroD1 and Neurog1, two transcription factors expressed in new neurons, were also reduced in the hippocampus of *Fmr1* KO mice (Figure S5, n = 3, p<0.05). Therefore, the loss of Fmrp leads to reduced neuronal differentiation but greater glial differentiation in aNPCs residing in the DG. These *in vivo* data along with our *in vitro* results suggest that Fmrp indeed plays important roles in regulating the differentiation and proliferation of aNPCs.

### Fmrp regulates the mRNAs of critical factors involved in aNPC proliferation and differentiation

**Figure 5:**
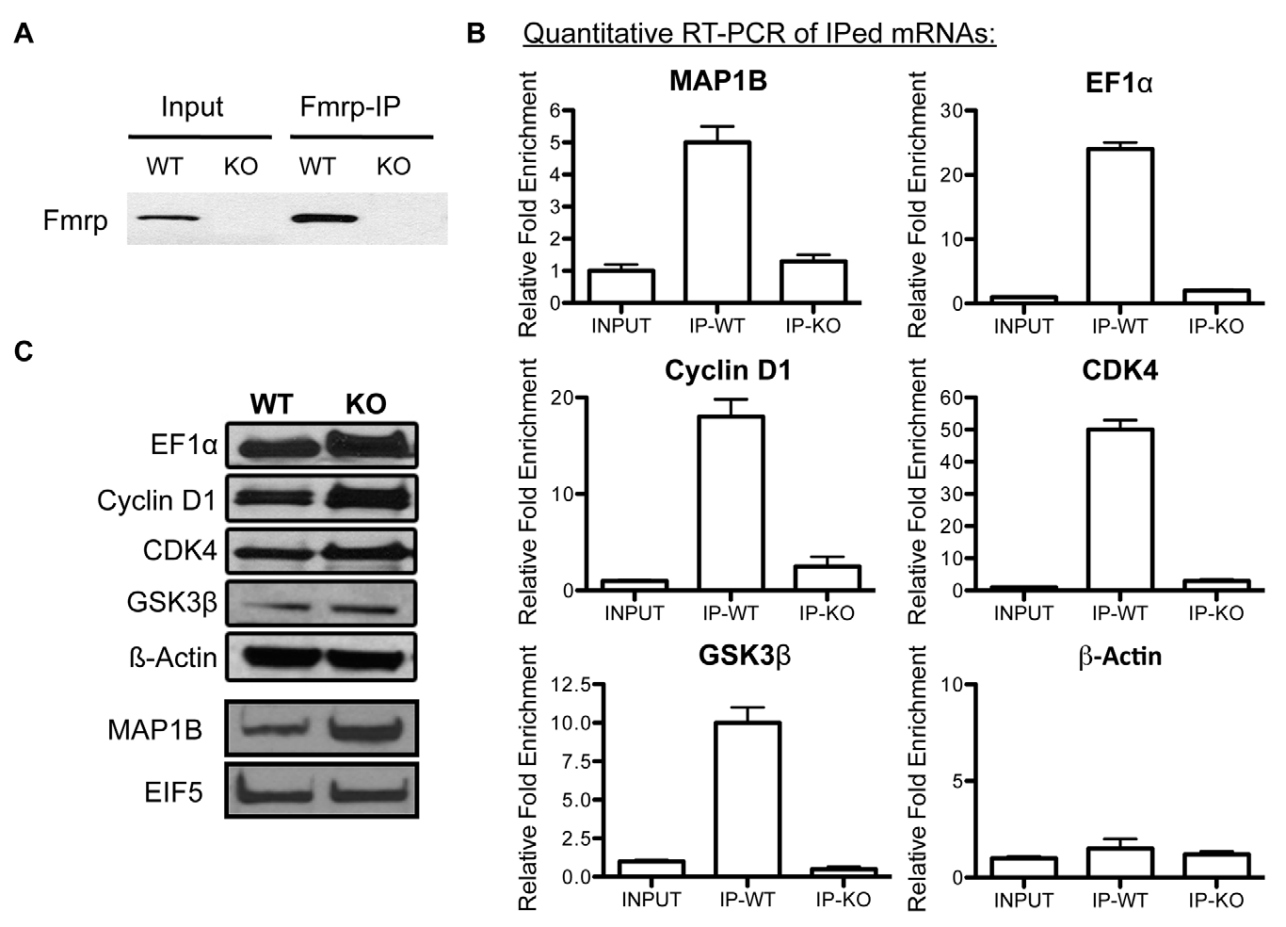
Identification of the mRNAs regulated by Fmrp in aNPCs. (A) Western blotting shows the amount of Fmrp in both input and immunoprecipitated Fmrp-containing mRNP complexes from both WT and *Fmr1* KO aNPCs. (B) The RNAs from Input and from Fmrp-IP of WT and KO cells were isolated and subjected to cDNA synthesis and real-time PCR quantification. The results confirmed that Fmrp binds to the mRNAs of MAP1B, EF1a, CDK4, cyclin D1, and GSK3β in WT aNPCs. KO aNPCs and ß-Actin mRNA analyses were used as negative controls. (C) Representative western blotting image showing the protein expression levels of the target genes of Fmrp in both WT and *Fmr1* KO aNPCs. EIF5 was used as a loading control for MAP1B, and ß-actin was used as a loading control for the others in western blots. Quantification of western blot band intensities is shown in Figure S6A.

As an RNA-binding protein, Fmrp is known to bind to a subset of specific mRNAs and suppress their translation [43]. To identify the mRNAs that are regulated by Fmrp in aNPCs, we employed the strategy of specifically immunoprecipitating Fmrp-containing mRNP particles and identifying the copurified mRNAs by probing expression microarrays, which we established previously [44]. Due to the large quantity of cells needed, we only used forebrain aNPCs derived from WT and *Fmr1* KO mice for immunoprecipitation with an antibody that could specifically precipitate Fmrp (Figure 5A). Both immunoprecipitated and input RNAs were used to probe Affymetrix arrays (data not shown). The mRNAs of interest were further confirmed to be associated with Fmrp by independent IP and real-time PCR (Figure 5B). Among these mRNAs, we found several already known to be regulated by Fmrp, such as MAP1B [2] and EF1α [3], confirming the specificity of our assay (Figure 5B and 5C). Also among the identified mRNAs, we found two key factors well established as enhancers of cell cycle progression, cyclin-dependent kinase 4 (CDK4) and cyclin D1. Their specific association with Fmrp was further confirmed by additional IP and RT-PCR (Figure 5B). We therefore examined the expression levels of CDK4 and cyclin D1 in both WT and *Fmr1* KO aNPCs. Though there was no significant change in the mRNA levels (Figure S6B), the loss of Fmrp led to higher protein levels of both genes (Figure 5C, Figure S6). Both CDK4 and cyclin D1 expression levels are important for the proliferation of neural progenitors [45],[46],[47],[48]. We found that a chemical inhibitor of CDK4 could partially rescue the proliferation phenotype of *Fmr1* KO aNPCs (Figure S6C). Hence increased expression of CDK4 and cyclin D1 as a result of Fmrp deficiency could be responsible for the increased proliferation of *Fmr1* KO aNPCs.

We also noticed that the mRNA of GSK3ß, known to be involved in the Wnt signaling pathway, could be coimmunoprecipitated with Fmrp from aNPCs. We confirmed the specific association between Fmrp and the mRNA of GSK3ß using additional Fmrp IP coupled to real-time PCR (Figure 5B). Furthermore, we confirmed that the loss of Fmrp led to increased protein levels of GSK3β (Figure 5C) and reduced protein levels of ß-catenin (Figure S7), a downstream target of GSK3β in proliferating *Fmr1* KO aNPCs.

### Loss of Fmrp alters the activity of the Wnt signaling pathway in adult neurogenesis

To determine whether Fmrp could regulate the translation of GSK3β protein, we cloned the 3′ untranslated region (3′UTR) of GSK3β and inserted it into the 3′ region of the Renilla luciferase coding sequence, such that the translation of Renilla luciferase could be regulated by the 3′UTR of GSK3β. Upon transfection of this construct into *Fmr1* KO and WT aNPCs, we observed significantly higher Renilla luciferase activity in *Fmr1* KO aNPCs compared with WT aNPCs, suggesting that the 3′UTR of GSK3ß leads to increased translational activity in *Fmr1* KO cells (Figure S7A). To further ensure that this increased protein level was due to increased translation rather than reduced protein stability of GSK3β in *Fmr1* KO cells, we treated *Fmr1* KO and WT aNPCs with the protein synthesis inhibitor cycloheximide over a 24-hour period. We found that, even though the GSK3β protein level was higher in the KO cells (time 0 h), there was no significant difference in the rate of GSK3β protein degradation between WT and KO aNPCs (Figure S7B). Therefore, these data suggest that Fmrp regulates the protein translation of GSK3β.

**Figure 6:**
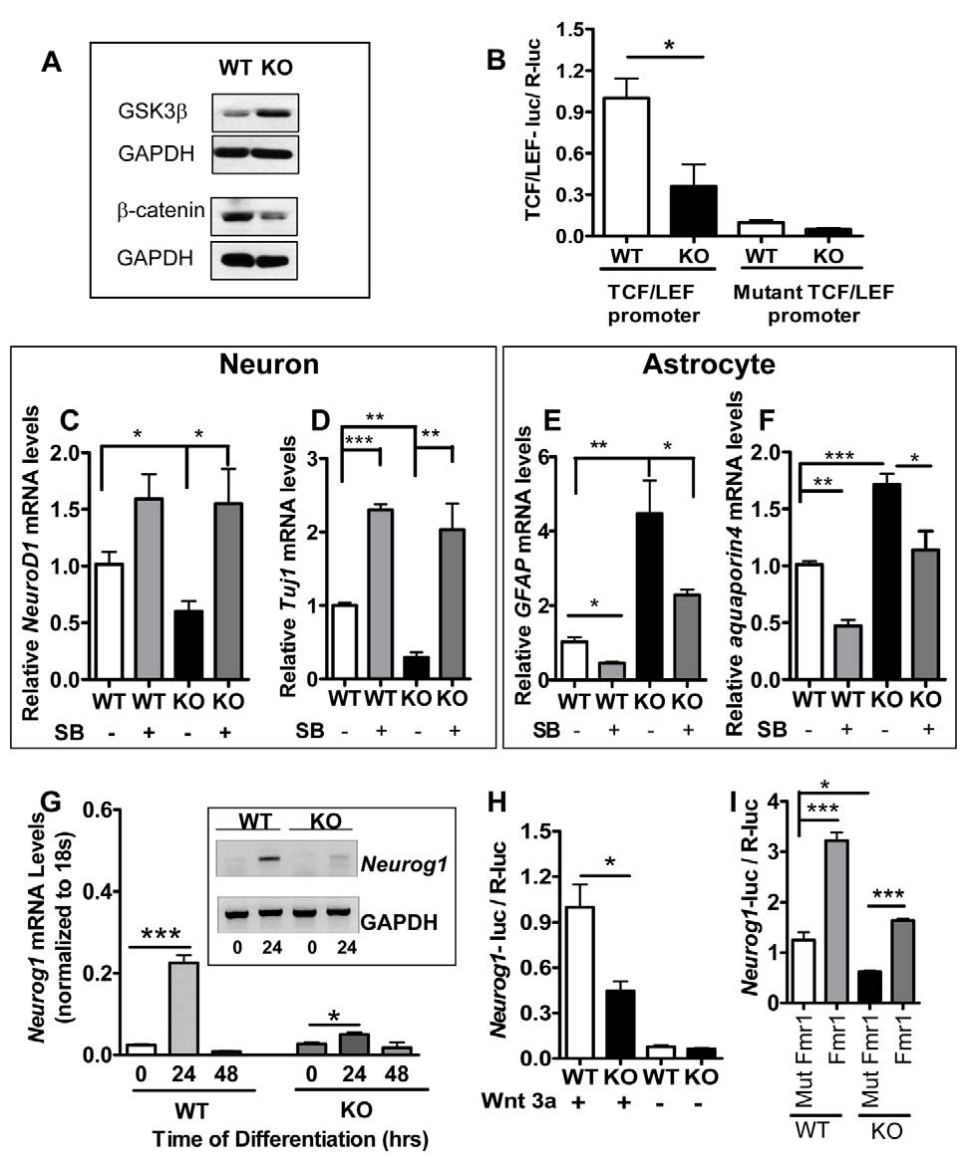
Loss of Fmrp leads to a deficit in the Wnt signaling pathway and reduced Neurog1 expression in aNPCs. (A) In differentiating *Fmr1* KO aNPCs (24 hours after initiation of differentiation), the GSK3β protein level was higher and β-catenin protein level was lower compared with differentiating WT aNPCs. (B) Differentiating *Fmr1* KO aNPCs have defective Wnt signaling, as indicated by the level of TCF/LEF-luciferase activity. A mutant promoter with the TCF/LEF site mutated was used as a negative control (n = 3). (C–F) The GSK3β inhibitor SB216763 (SB) could partially rescue the reduced neuronal (C,D) and increased astrocyte (E,F) differentiation deficits of *Fmr1 KO* aNPCs. SB (dissolved in DMSO) was added at initiation of differentiation at 4 µM. An equal amount of DMSO was added to WT and KO control aNPCs. Cell differentiation was assessed by the relative mRNA levels of *NeuroD1* (C), *Tuj1* (D), *GFAP* (E), and *aquporin4* (F). GAPDH mRNA levels were used as an internal control. (G) Real-time quantitative PCR results show that early differentiating (24 hours) WT aNPCs transiently express high levels of Neurog1 (∼10-fold induction compared with 0 hour; n = 4). This Neurog1 induction is drastically impaired in differentiating (24 hours) *Fmr1* KO aNPCs (<2×-fold; n = 4). Proliferating aNPCs (0 hour), and later differentiating (48 hours) cells, expressed a minimal level of *Neurog1*. Inset, similar results obtained by regular RT-PCR. (H) The Wnt receptor ligand, Wnt3a, is required for activating the *Neurog1* promoter during differentiation. In the presence of Wnt3a, *Neurog1* promoter activity was significantly lower in *Fmr1* KO aNPCs compared with WT cells. *Neurog1* promoter activity was undetectable in the absence of Wnt3a (n = 3). (I) Exogenously expressed wild-type Fmr1, but not mutant Fmr1, could promote the *Neurog1* transcription as assessed by *Neurog1* promoter activities in both *Fmr1* KO and WT aNPCs (n = 3). All data are shown as mean ± SEM, and Student's t-test was used for all the analyses. *, p<0.05, **, p<0.01***, p<0.001.

The canonical Wnt pathway is known to be critical for adult neurogenesis, but the downstream effectors have been a mystery [20],[26]. Since Fmrp was able to regulate the translation of GSK3β, we further investigated whether the activity of the Wnt pathway was altered in *Fmr1* KO aNPCs. GSK3β is known to phosphorylate and promote the proteasome degradation of ß-catenin, a central player in the Wnt signaling pathway. We therefore chose to examine the expression of ß-catenin in aNPCs. In both proliferating and differentiating aNPCs, we observed increased GSK3β protein levels (Figure 5C) and decreased expression of ß-catenin (Figure 6A and Figure S7C). Hence Fmrp may promote adult neurogenesis by regulating the expression of GSK3β and subsequently ß-catenin.

In the absence of Wnt, ß-catenin is known to be held in cytosol and degraded by a collection of regulatory factors, such as GSK3β [27]. The activation of Frizzled by Wnt leads to stabilization and nuclear translocation of ß-catenin, which forms a complex with TCF/LEF transcription factors and induces the expression of downstream target genes [27]. To confirm that loss of Fmrp led to the deficit in the Wnt signaling pathway, we used a well-characterized luciferase reporter system for monitoring the activity of the Wnt signaling pathway [26],[49]. Upon growth factor withdrawal and activation by cotransfected Wnt3a expression vector, *Fmr1* KO aNPCs exhibited significantly reduced luciferase activity compared with WT aNPCs (Figure 6B). In addition, expression of Axin2, a downstream effector of the Wnt signaling pathway, was reduced in the hippocampus of *Fmr1* KO mice (Figure S8). Therefore, the Wnt signaling pathway is indeed defective in the absence of Fmrp. In addition, treatment of *Fmr1* KO aNPCs with a well-established GSK3β inhibitor SB216763 [50] could enhance the Wnt signaling pathway () and partially rescue the neuronal (Figure 6C and 6D) and astrocyte (Figure 6E and 6F) differentiation deficits in aNPCs. Similar results were also obtained using the DG aNPCs (Figure S9B and S9C). Interestingly, SB216763 also repressed aNPC proliferation without affecting cyclin D1 expression levels (Figure S10). Therefore, Fmrp deficiency leads to reduced Wnt signaling, which could be responsible for altered aNPC differentiation.

### Loss of Fmrp alters the expression of Neurog1 in aNPCs

The basic helix-loop-helix family transcription factor neurogenin1 (Neurog1) can be regulated by Wnt signaling, and its promoter contains one single classic TCF/LEF binding element [51]. We therefore assessed the mRNA levels of *Neurog1* in *Fmr1* KO proliferating and differentiating aNPCs. *Neurog1* was transiently expressed in differentiating WT aNPCs (Figure 6G), as shown previously [51]. We found that *Neurog1* mRNA levels indeed decreased in *Fmr1* KO differentiating aNPCs (Figure 6G). To determine whether the altered *Neurog1* expression resulted from a Wnt signaling deficit in *Fmr1* KO aNPCs, we created a reporter construct that has a mouse native *Neurog1* promoter driving the expression of luciferase. When transfected into *Fmr1* WT and KO aNPCs that were subjected to differentiation, the *Neurog1*-luciferase reporter yielded detectable luciferase activity only in the presence of Wnt3a (Figure 6H), indicating that this promoter is activated by Wnt signaling. As expected, we found that *Neurog1* promoter activity was significantly reduced in differentiating *Fmr1* KO aNPCs compared with WT cells (Figure 6H). Furthermore, we could rescue the *Neurog1* promoter activity by expressing the wild-type but not the mutant Fmr1 in *Fmr1* KO aNPCs (Figure 6I). Taken together, these data suggest that the expression of Neurog1 is controlled by Fmrp through the Wnt signaling pathway in aNPCs.

**Figure 7:**
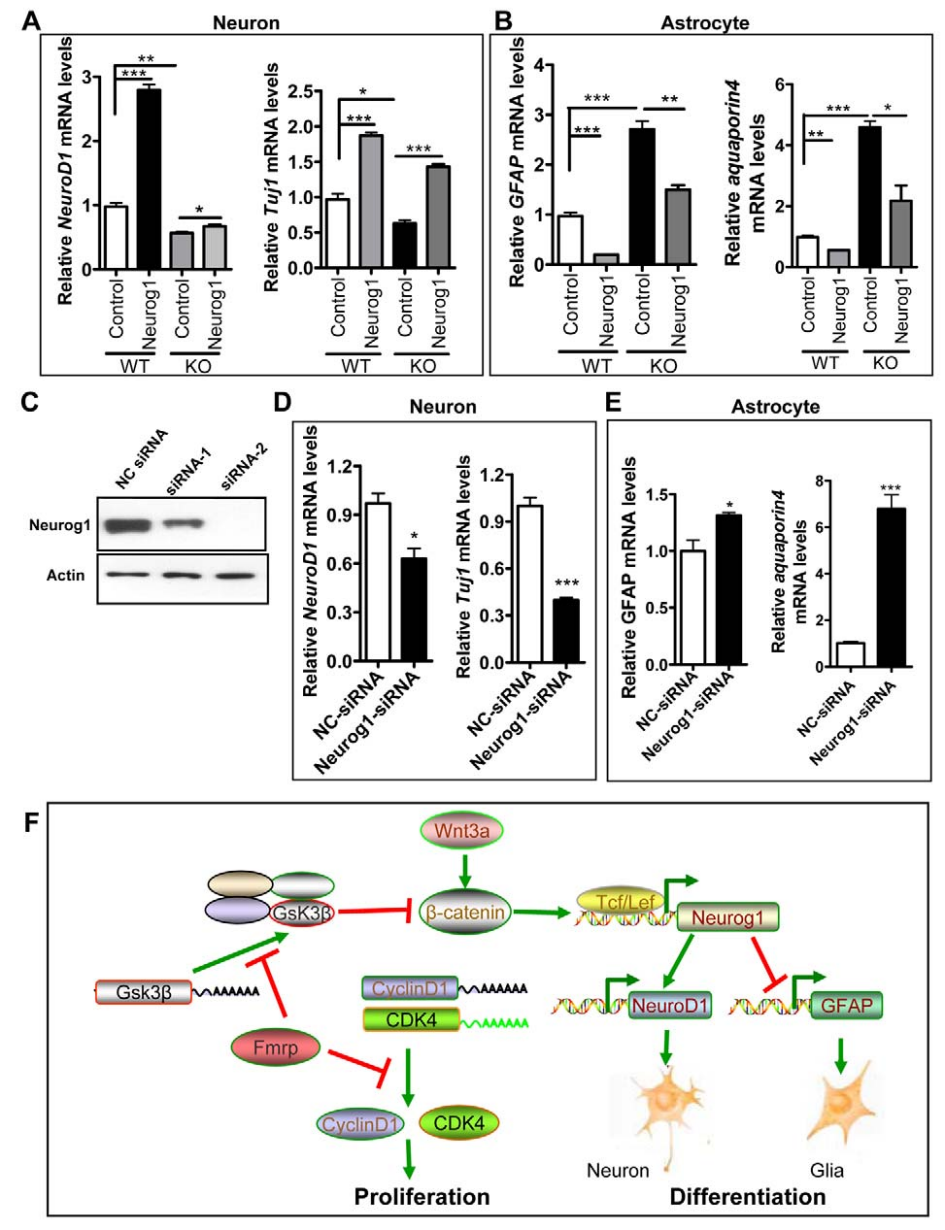
Neurog1 regulates the fate specification of aNPCs. (A,B) Exogenously expressed Neurog1 could rescue the neuronal and astrocyte differentiation deficits of *Fmr1* KO aNPCs, as assessed by real-time PCR of neuron (*NeuroD1* and *Tuj1*) and astrocyte (*GFAP* and *aquaporin4*)-specific gene expression (n = 3; Control, pCDNA3 empty vector) (C) Neurog1-siRNA could specifically reduce the Neurog1 protein expression from a co-transfected Neurog1 expression vector. siRNA-2 was more effective at reducing Neurog1 protein expression, and was therefore used in all functional tests. NC-siRNA: Nonsilencing Control siRNA. (D,E) Acute knockdown of Neurog1 expression in aNPCs led to reduced neuronal differentiation (D), but increased astrocyte differentiation (E) in WT aNPCs, as assessed by real-time PCR of cell lineage-specific genes (n = 3). Cell differentiation was assessed by the relative mRNA levels of *NeuroD1* (A and D, left), *Tuj1* (A and D, right), *GFAP* (B and E, left), and *aquporin4* (B and E, right). GAPDH mRNA levels were used as an internal control for all real-time PCR analyses, unless stated otherwise. (F) Model of Fmrp functions in adult neurogenesis. By regulating the translation of cyclin D1 and CDK4, Fmrp controls the proliferation of aNPCs. By controlling the translation of GSK3β, Fmrp maintains the proper intracellular levels of β-catenin and Wnt signaling. Upon differentiation, β-catenin positively regulates the expression of Neurog1, which promotes neuronal differentiation and represses glial differentiation. All data are shown as mean ± SEM, and Student's t-test was used for all the analyses. *, p<0.05; ***, p<0.001.

Since Neurog1 is an early initiator of neuronal differentiation and an inhibitor of glial differentiation [28], its downregulation could be responsible for the reduced neuronal differentiation and increased glial differentiation seen in *Fmr1* KO aNPCs. To test this possibility, we expressed exogenous Neurog1 in *Fmr1* KO forebrain aNPCs and found that exogenously expressed Neurog1 could rescue the altered fate specification of *Fmr1* KO aNPCs, as assessed by the mRNA levels of neuronal genes (Figure 7A, *NeuroD1* and *Tuj1*) and astrocytic genes (Figure 7B, *GFAP* and *aquqporin4*), as well as the promoter activity of *NeuroD1* and *GFAP* (data not shown) in differentiating cells. To further validate the role of Neurog1 in aNPC differentiation, we acutely knocked down Neurog1 expression in aNPCs using siRNA (Figure 7C) and found that acute knockdown of Neurog1 in aNPCs led to decreased neuronal differentiation (Figure 7D), but increased astrocyte differentiation (Figure 7E), reminiscent of what we found in *Fmr1* KO aNPCs. Similar results were also obtained using the DG aNPCs (Figure S11). Therefore, our findings suggest that Fmrp regulates aNPC fate specification by modulating the activity of the Wnt/β-catenin signaling pathway and subsequently its downstream effector, Neurog1 (Figure 7F).

## Discussion

In this study we demonstrate that the loss of functional Fmrp in aNPCs leads to reduced neurogenesis both *in vitro* and *in vivo*. We show that Fmrp regulates the translation of several factors involved in stem cell proliferation and differentiation, including CDK4, cyclin D1, and GSK3β. As a result of dysregulation of GSK3β and the Wnt signaling pathway, the expression level of *Neurog1*, one of the Wnt-regulated genes, is reduced, which is likely responsible for the reduced neuronal differentiation and increased astrocyte differentiation seen in *Fmr1* KO aNPCs. Our data demonstrate that Fmrp plays profound regulatory roles in adult neurogenesis.

Despite exhaustive efforts devoted to understanding transcriptional regulation in adult neurogenesis, the role of translational control in adult neurogenesis has gone largely unexplored; yet our results indicate that translational control is just as important, if not more so, in the regulation of aNPC functions. We have identified the molecular pathways by which Fmrp regulates aNPC proliferation and fate specification. Both a previous study from another group [52] and our current study found that the mRNAs of both CDK4 and cyclin D1 could be bound by Fmrp. CDK4 and cyclin D1 are well-characterized cell-cycle regulators in many cell types [53]. In mammalian neural progenitor cells, increased cyclin D1 expression is positively correlated with their proliferation [54], and reduced cyclin D1 levels result in decreased proliferation [48]. CDK4 has been shown to regulate the proliferation of neural progenitors in adult brains [47], and inhibition of CDK4 activity leads to growth arrest in neural progenitors [46]. The fact that we could rescue the proliferation deficits of *Fmr1* KO aNPCs using a chemical inhibitor of CDK4 supports our model that Fmrp regulates aNPC proliferation in part through CDK4.

We also found here that Fmrp could bind and regulate the translation of GSK3β mRNA. As a negative regulator of the Wnt signaling pathway, GSK3β promotes the degradation of β-catenin and inhibits the activity of the canonical Wnt signaling pathway [27]. The Wnt signaling pathway has been shown to promote the proliferation of a number of cell types, including hematopoietic stem cells [55]. Although one study suggests that Wnt signaling can also promote cell proliferation in the DG [56], other publications clearly point out the function of the Wnt signaling pathway in activating neuronal differentiation during adult neurogenesis, and inhibiting this pathway results in hippocampus-dependent learning deficits [20],[26],[57]. Our data show *Fmr1* KO aNPCs had reduced Wnt signaling, and we identified Neurog1 as one of the downstream targets of Fmrp and Wnt. Neurog1 is a transcription factor expressed only at the early stage of differentiation, and it promotes neuronal differentiation while inhibiting astrocyte differentiation [28],[29]. Neurog1 contains a conserved Tcf/Lef binging site in its promoter, allowing it to sense the levels of Wnt signaling. Although the Wnt signaling pathway has been found to enhance cyclin D1 transcription in HeLa cells and several other cell types [58], we saw no such activation in aNPCs. Interestingly, enhancing Wnt signaling via a Gsk3β inhibitor repressed proliferation of *Fmr1* KO aNPCs, possibly due to the neuronal differentiation effect of Wnt signaling. It is likely that in aNPCs, Wnt signaling and cyclin D1 act independently on cell proliferation, and they are both also regulated by Fmrp.

Several studies have examined embryonic and early postnatal neurogenesis in mice [25] and humans [24]. One study found that the loss of Fmrp led to increased neuronal differentiation and reduced glial differentiation in mice [25]. Due to the large scale of embryonic neurogenesis, factors affecting aNPCs would also be expected to affect both the overall number of neurons, as well as brain size. However, neither adult fragile X patients nor adult *Fmr1* KO mice show any differences in the number of neurons and glia compared with controls [59], raising questions about the potential significance of increased early neurogenesis to the pathogenesis of fragile X syndrome. Another study found no alteration in the differentiation of embryonic NPCs (eNPCs) isolated from one human embryo diagnosed with a fragile X mutation [24]. While the discrepancies between human and mouse studies require further confirmation using additional human tissues, the different phenotypes observed in Fmrp-deficient eNPCs versus aNPCs support the idea that adult neurogenesis is subjected to regulatory mechanisms distinct from those in embryonic neurogenesis [22]. First, during adult neurogenesis, multipotent aNPCs are in intimate contact with the surrounding mature neurons and glia, and the fate of aNPCs can be affected by their microenvironment [8],[9],[22],[60]. Mice that lack Sonic hedgehog [61], Tlx [62], Bmi1 [63],[64], and Mbd1 [33] have all exhibited profound deficits in postnatal neurogenesis, but not in their embryonic neural development. In fact, in prenatal and early postnatal developing brains, Fmrp is widely expressed in neural cells, including glia and glial precursors, with the levels of Fmrp decreasing during oligodendrocyte differentiation [65],[66], whereas in adult brains, Fmrp is expressed predominantly in neurons, with negligible expression in mature glia [30],[31]. Further studies into the role of Fmrp in both embryonic and adult neurogenesis would facilitate our understanding of the unique molecular networks that regulate eNPCs and aNPCs at the level of translational control.

Hippocampal neurogenesis has been associated with hippocampus-dependent learning [8],[9], and blocking neurogenesis using methods nonexclusive to adult NPCs or new neurons has supported this model [14]–[17],[20]. Altered adult hippocampal neurogenesis and impaired learning have been found in several pathological conditions [9],[21]; however, the possibility of a link between adult neurogenesis and human mental retardation disorders, though recently put forward [67], has not been studied well. Although there is a low level of DG neurogenesis in adults, mounting evidence points to its potentially important role in neuroplasticity, emotional behavior, and the higher cognitive functions of adult brains. It has been proposed that adult neurogenesis enables the lifelong adaptation of the hippocampal network to the levels of novelty and complexity a person experiences [11]. Using precise and unbiased stereological methods coupled with confocal microscopy, we observed a mild but significant reduction in the number of new neurons in *Fmr1* KO mice, which could easily have been missed by others who employed non-stereology quantification methods [68]. Due to the restricted nature and low level of adult neurogenesis, a lack of Fmrp may not affect the total number of neurons in adult brains, but it can contribute to pathological conditions linked to higher cognitive functions and learning abilities [9],[67]. The learning deficits of the *Fmr1* KO mice may be the result of both reduced neurogenesis and defective neuronal maturation. Consistent with the literature [69], we have observed that *Fmr1* KO aNPC-differentiated neurons had reduced dendritic complexity and length (data not shown), which could also contribute to behavioral deficits. In addition, although *Fmr1* KO mice have increased proliferation, at four weeks post-BrdU labeling, both KO and WT mice had similar numbers of surviving new cells, possibly due to the decreased survival of new cells in KO mice. How Fmrp regulates the survival of young neurons is another interesting question that is currently being pursued as an independent study. One mystery that remains to be cleared up is why the size of the DG in the adult *Fmr1* KO mice is bigger than in controls. Since the new cells generated in the adult DG account for only a small portion of the total DG cells, increased proliferation of these new cells may not contribute much to the increased size of the DG. Castren et al. [25] have shown that *Fmr1* KO mice exhibit increased cell proliferation in the subventricular zone during embryonic development (E13). The mammalian DG is formed during the postnatal period, with P7 as the peak of cell genesis. It is possible that increased cell proliferation during DG formation results in an increased DG volume that persists into adulthood. In addition to its function in the initial stage of neurogenesis, Fmrp-deficient neurons are known to have reduced dendritic complexity [25],[70]; therefore, it is possible that new neurons generated in the adult DG also have reduced dendritic complexity. Hence deficits in several stages of adult neurogenesis could contribute to the higher brain functions, such as the learning and emotional disabilities associated with fragile X patients, without significantly affecting the gross brain structure of human patients.

Our results suggest that translational regulation by Fmrp in aNPCs and young neurons is essential for learning and memory, and the reduced number of new neurons together with defective maturation of these new neurons may contribute to the cognitive deficiency seen in fragile X patients. This is a facet of the etiology of fragile X syndrome that has not been recognized before.

## Materials and Methods

### Fmr1 KO mice

All animal procedures were performed according to protocols approved by the University of New Mexico Animal Care and Use Committee. The *Fmr1* KO mice bred onto the C57B/L6 genetic background were as described previously [71].

### Isolation and cultivation of adult NPCs

Adult aNPCs used in this study were isolated from 8- to 10-week-old male *Fmr1* KO mice and wild-type (WT) controls based on published methods: for the forebrain aNPCs [33] and for the DG aNPCs [72]. (See Text S1 for details.)

### Proliferation, differentiation, cell death analyses, and chemical treatment of cultured aNPCs

These analyses were carried out using our established method [32],[34]. (See Text S1 for details.)

### *In vivo* neurogenesis studies

*In vivo* neurogenesis analyses were performed essentially as described previously [32],[33]. These experiments have been performed using 3 different batches of animals, with n = 4–6/genotype each batch. For the first two batches, BrdU (50mg/kg) was injected into 8-week-old mice daily for 7 consecutive days to increase the amount of labeling. Mice were then euthanized 1 day post-injection to assess the *in vivo* proliferation (and early survival) of labeled cells. For cell survival analysis, another group of mice was injected with BrdU at 8 weeks of age and euthanized 4 weeks post-injection. The third batch of mice, on the other hand, were given 6 injections of BrdU (50 mg/kg) within 24 hours to label all dividing cells in the DG within this time period and sacrificed at 4 hours post-last injection based on a published protocol [41]. Mice were euthanized by intraperitoneal injection of sodium pentobarbital, and then transcardially perfused with saline followed by 4% PFA. Brains were dissected out, post-fixed overnight in 4% PFA, and then equilibrated in 30% sucrose. Forty-µm brain sections were generated using a sliding microtone and stored in a −20°C freezer as floating sections in 96-well plates filled with cryoprotectant solution (glycerol, ethylene glycol, and 0.1 M phosphate buffer, pH 7.4, 1∶1∶2 by volume). We performed immunohistological analysis on 1-in-6 serial floating brain sections (240 µm apart) based on the published method [33]. (Please see Text S1 for more details.)

### DNA plasmids

The DNA plasmids carrying 2.5 kb of glial fibrillary acidic protein (*GFAP*) promoter-firefly luciferase reporter gene (GF1L-pGL3) or its mutant version, with the STAT3 binding site mutated (GF1L-S-pGL3), and an internal control plasmid containing sea pansy luciferase driven by human elongation factor 1α promoter (EF1α-Luc) were as described previously [34],[73]. NeuroD1-luciferase, a gift from Dr. F.H. Gage, was then cloned into pGL3 plasmid. *Fmr1*-siRNA, control-siRNA, and mouse Neurog1 expression vector were purchased from Open Biosystems (www.openbiosystems.com). Neurog1 siRNA was purchased from SABiosciences (Frederick, MD). Wild-type Wnt reporter construct pTOPFLASH containing 8 TCF/LEF binding sites and mutant reporter construct pFOPFLASH were gifts from R.T. Moon (University of Washington) as described [26]. Wnt3a expression plasmid was a gift from Dr. D.C. Lie (Institute of Developmental Genetics, Germany) as described [26]. Wild-type FLAG-Fmrp was cloned into pDEST-27 vector, and mutant FLAG-I304N was generated by site-directed mutagenesis (Stratagene) [40]. All the constructs were verified by DNA sequencing. Myelin basic protein (MBP) promoter was cloned from mouse genomic DNA based on published information [74] and cloned into pGL3 plasmid. The mouse *Neurog1* promoter, containing its native TCF/LEF binding site “cctttgaa,” was cloned by PCR based on the GenBank sequence (GenBank ID #18014) using the following primers: 5′-GTCTGACTCTGAAGCCATCTCTGA-3′ (forward) and 5′ -ACGCGCCGGGCTGGTCTCCT-3′ (reverse). The PCR product was then subcloned into the pCRII-TOPO plasmid, sequenced, and inserted into the *KpnI-XhoI* site of the pGL2 basic vector to yield *Neurog1*-luciferase reporter construct. The full-length 3′-UTR of GSK-3ß mRNA was PCR-amplified directly from proliferating aNPC first-strand cDNA generated from 5 µg TRIzol-isolated total RNA using oligo-dT SuperScript III reverse transcription according to the manufacturer's protocol (Invitrogen, Cat. #1808-093). It was cloned into pIS2 Renilla luciferase vector, and pIS0 firefly luciferase was used as a transfection control [75].

### Electroporation, transfection, and luciferase assay

Electroporation of plasmid DNA into aNPCs and the luciferase assay were carried out using an Amaxa Nucleofector electroporator based on the manufacturer's protocol (Amaxa, #VPG-1004) with modifications [34]. Briefly, 2×10^6^ cells were trypsinized, resuspended in Nucleofector solution, mixed with DNA, and electroporated using a preset program for mouse NPCs (#A033). The cells were then plated onto polyornithin/laminin-coated 24-well plates in proliferation medium. After 24 h, cells were changed into differentiation medium for 48 h. Transfection of aNPCs was carried out using Stemfect (Stemgent, San Diego, CA) based on the manufacturer's protocol with modifications. Briefly, aNPCs were plated into 24-well P/L-coated plate for 24 hours. Then 3 µg DNA and 0.9 µl Stemgent reagent were mixed, incubated for 10 minutes, and then added to the cells. Sixteen hours later, the transfected cells were changed into differentiation medium for 48 hours. The cells were then collected and luciferase activity was detected using the Dual-Luciferase Reporter 1000 System (Promega, Cat# E1980) based on the manufacturer's protocol. Briefly, collected cells were lysed in 100 µl of 1× passive lysis buffer at room temperature for 15 minutes. Then 20 µL of the lysate was added to 100 µl of Luciferase Assay Buffer II and mixed briefly. Firefly luciferase (F-luc) activity was immediately read using a SpectraMax M2E plate reader (Molecular Devices Corp.). Next, 100 µl of Stop & Glo Buffer with Stop & Glo substrate was added and mixed briefly. Renilla luciferase (R-luc) activity was immediately read. F-luc activity was normalized to R-luc activity to account for variation in transfection efficiencies. Each experiment was independently repeated 3 times. For each electroporation, 3 µg (*NeuroD1*− or *GFAP*−) luciferase DNA, 5 µg *Neurog1*-luciferase DNA, 0.2 µg R-Luc, and 0.004–2 µg *Fmr1*, Neurog1, or control expression plasmids were used.

### RNA immunoprecipitation, microarray assay, and real-time PCR

These procedures were carried out as described [43]. (Please see Text S1 for details.)

### Western blots

Twenty-mg protein samples were separated on SDS-PAGE gels and then transferred to PVDF membranes (Millipore). Membranes were processed following the ECL western blotting protocol (GE Healthcare). anti-MAP1B (a gift from I. Fischer, Drexel University, Philadelphia), anti-Nestin (Millipore), anti-Fmrp (7G1-1), anti-Fmrp (John Louis), anti-β-catenin (Millipore), anti-CDK4 (Millipore), anti-Cyclin D1 (Upstate), anti-TCF4 (Abcam), GSK3ß (Abcam), anti-EF1α (ATCC), anti-Neurog1 (Millipore), anti-NeuroD1 (Santa Cruz), anti-Axin2 (Cell Signaling) and anti-β-Actin (Abcam) were used as primary antibodies at the concentrations recommended by the manufacturers. HRP-conjugated secondary antibodies were obtained from Sigma. For loading controls, membranes were stripped and reprobed with the antibody against eIF5α (Santa Cruz Biotechnology), anti-GAPDH (Ambion), or eIF4E (Transduction Laboratories). To test the efficiency of *Fmr1*-siRNA, Fmrp expression plasmid and siRNA expression plasmid were cotransfected into HEK293 cells, and the mRNA and protein expression levels of Fmrp were analyzed using PCR and western blot, respectively.

### Statistical analysis

Statistical analysis was performed using ANOVA and Student's t-test, unless specified with the aid of SPSS v.17. All data were shown as mean with standard error of mean (mean ± SEM). Probabilities of P<0.05 were considered significant.

## Supporting Information

Figure S1 Fmrp is expressed in DG neurons but not astrocytes in the adult hippocampus. (A) Fmrp staining is prominent in the majority of the DG cells of WT mice but is absent in the KO mice. (B,C) Fmrp expression was nearly undetectable in GFAP (B) or S100β (C) expressing astrocytes. Arrows point to astrocyte that are negative for Fmrp staining. Scale bars = 10 µm.
(1.47 MB PDF)

Figure S2 Adult brain-derived aNPCs from *Fmr1* KO mice exhibited altered proliferation. (A) Single plain Laser Scanning Confocal image showing that adult brain-derived aNPCs cultured under proliferating conditions expressed neural progenitor markers: Nestin (cytoplasmic, red) and Sox2 (nuclear, green). Dapi was used to label nuclear DNA (blue). (B–E) Cell cycle profile of WT and *Fmr1* KO aNPCs indicating that Fmr1 KO aNPCs had more cells in mitosis (G2/M phase) and fewer cells in S phase. N = 3 independent cell preparations. *, p<0.05, Student's t-test. Data is shown as mean ± SEM.
(0.09 MB PDF)

Figure S3 Fmr1-siRNA could specifically reduce the mRNA and protein expression of Fmrp as shown by real-time PCR (A) and Western blotting (B).
(0.04 MB PDF)

Figure S4 aNPCs isolated from the DG of *Fmr1* KO mice had similar phenotypes as those found in aNPCs isolated from the *Fm1* KO forebrain. (A,B) *Fmr1* KO DG aNPCs exhibited lower *NeuroD1* promoter (A) but higher *GFAP* promoter (B) activities. (C,D) *Fmr1* KO DG aNPCs had lower levels of endogenous NeuroD1 mRNA (C) but higher levels of endogenous *GFAP* mRNA (D). (E–H) Acute knockdown of Fmrp expression in WT DG aNPCs using siRNA led to decreased neuronal promoter activity (E; mean ± SEM n = 6, p<0.05) and decreased *NeuroD1* mRNA levels (F), but increased *GFAP* promoter activity (G; mean ± SEM n = 6, p<0.05) and increased *GFAP* mRNA levels (H; p<0.001). Therefore, Fmrp has similar functions in DG aNPCs compared to aNPCs derived from the forebrain. All data are shown as mean ± SEM. Statistics was done using two tailed unpaired Student's t-test. *, p<0.05; **, p<0.01; ***, p<0.001. NC-siRNA, nonsilencing control siRNA.
(0.10 MB PDF)

Figure S5 Reduced expression of NeuroD1 and Neurogenin1 in *Fmr1* KO mice (A,B). The protein levels of two transcription factors specific to young neurons, NeuroD1 (A) and Neurog1 (B), exhibited lowered expression levels in Fmr1 KO hippocampus, as assessed by Western blot analysis. Sample images of Western blots are shown in the upper panels and quantification of 3 blots are shown in the lower panels. β-actin was used as a loading control. (C) Immuno histological staining using shows reduced number of NeuroD1-positive Cells (white arrows) in the subgranular zone of the DG. All data are shown as mean ± SEM. Statistics were done using two tailed unpaired Student's t-test. *,p<0.05; Scale bar = 10 µm.
(0.38 MB PDF)

Figure S6 Expression analysis of proliferating *Fmr1* KO aNPCs. (A) Quantification of Western blot band intensities (as shown in Figure 4C) normalized to ß-actin levels demonstrates increased protein levels of EF1a, CyclinD1, CDK4, GSK3β, and MAP1b in *Fmr1* KO aNPCs. Data is from n = 3 or 4 independent measurements with KO levels normalized to the WT levels. Student's t-test was performed on data before normalization to ensure accurate statistical analysis. (B) The mRNA levels of *EF1a*, *CyclinD1*, *CDK4*, *GSK3β*, and *MAP1b* were not changed in proliferating *Fmr1* KO aNPCs. The steady-state mRNA level determined by real-time PCR was normalized to18S. (C) CDK4 inhibitor was dissolved in DMSO (0 concentration). At 60 nM, this inhibitor can reverse the proliferation of *Fmr1* KO aNPCs and bring it to the level of WT cells (n = 3), suggesting that increased CDK4 activity might be a reason for increased proliferation of *Fmr1* KO aNPCs. Proliferation was assessed by BrdU pulse labeling followed by immunostaining and stereological quantification. All data are shown as mean ± SEM. Statistics were done using two tailed unpaired Student's t-test. *, p<0.05.
(0.14 MB PDF)

Figure S7 Fmrp regulates translation of GSK3β. (A) A *GSK3β* 3′untranslated region (3′UTR) was cloned into a Renilla luciferase (R-luc) expression vector (top panel) therefore the translation of R-luc was regulated by the 3′UTR of *GSK3β*. Transfection of this construct into aNPCs resulted in higher R-Luc activity (normalized to firefly luciferase internal control) in *Fmr1* KO compared with WT cells (Data is shown as mean ± SEM; n = 3, p<0.001, Student's t-test), suggesting that elevated translational activity is directed by GSK3β 3′UTR in the absence of Fmrp. Data is shown as mean ± SEM. Statistics were done using two tailed unpaired Student's t-test. ***, p<0.001. (B) aNPCs were treated with a protein synthesis inhibitor, cycloheximide, during a 24 hour period. Gsk3β protein levels were determined using Western blot (top panel) and quantified. The result indicates that the degradation rate of GSK3β protein is not significantly different between Fmr1 KO and WT aNPCs. (C) β-catenin protein expression was decreased in proliferating *Fmr1* KO aNPCs. PDF (35KB)
(0.13 MB PDF)

Figure S8 Reduced expression of Axin2 protein in the hippocampus of *Fmr1* KO mice The protein levels of Axin2, a downstream effecter of canonical Wnt signaling pathway, exhibited lowered expression levels in *Fmr1* KO hippocampus. Sample images of Western blots (left) and quantification of 3 blots (right) are shown. β-actin was used as a loading control. Data is shown as mean ± SEM. Statistics were done using two tailed unpaired Student's t-test. *, p<0.05.
(0.04 MB PDF)

Figure S9 Gsk3β inhibitor could rescue the neuronal and astrocyte differentiation deficits of *Fmr1* KO DG aNPCs. (A) Gsk3β inhibitor SB216763 (SB) SB could enhance the Wnt signaling in both WT and *Fmr1* KO aNPCs. (B,C) SB could rescue the reduced *NeuroD1* (A) mRNA levels and increased *GFAP* mRNA levels (B) in *Fmr1* KO aNPCs. SB (dissolved in DMSO) was added at initiation of differentiation at 4 µM. Equal amount of DMSO was added to WT and KO control aNPCs. All data are shown as mean ± SEM. Statistics were done using two tailed unpaired Student's t-test. *, p<0.05; **, p<0.01; ***, p<0.001.
(0.05 MB PDF)

Figure S10 Gsk3β inhibitor could reverse the proliferation deficit of *Fmr1* KO aNPCs. (A) Gsk3β inhibitor SB216763 (SB) SB could repress proliferation of *Fmr1* KO aNPCs. Effect on WT cells was not statistically significant (p = 0.08). (B) SB treatment did not affect cyclin D1 expression levels in either WT or KO aNPCs (n = 3). All data are shown as mean ± SEM. Statistics were done using two tailed unpaired Student's t-test. *, p<0.05; **, p<0.01. PDF (53KB)
(0.13 MB PDF)

Figure S11 Neurog1 regulates the fate specification of the DG aNPCs. Neurog1 could rescue the neuronal (A) and astrocyte (B) differentiation deficits of *Fmr1* KO DG aNPCs. Acute knockdown of Neurog1 in WT DG aNPCs led to reduced neuronal (C) but increased astrocyte (E) differentiation. NeuroD1 is an neuronal lineage marker. *GFAP* is an astrocyte lineage marker. The relative mRNA levels were in comparison with GAPDH mRNA. Promoter activities of NeuroD1 and *GFAP* (fire fly luciferase, luc) were normalized to a cotransfected internal control (E1a-Renilla luciferase, Rluc). All data are shown as mean ± SEM. Statistics were done using two tailed unpaired Student's t-test. *, p<0.05; **, p<0.01; ***, p<0.001.
(0.10 MB PDF)

Text S1 Supplemental methods.
(0.13 MB PDF)
