# Supplementary figures and images for: Fragile X Mental Retardation Protein Regulates Proliferation and Differentiation of Adult Neural Stem/Progenitor Cells
Source: PLoS Genet. 2010 Apr 8;6(4):e1000898. doi: 10.1371/journal.pgen.1000898 (PMC2851565; doi:10.1371/journal.pgen.1000898)

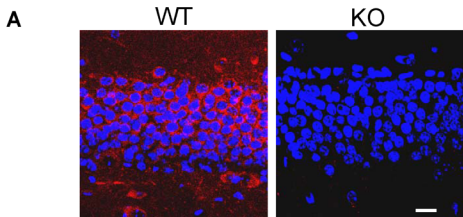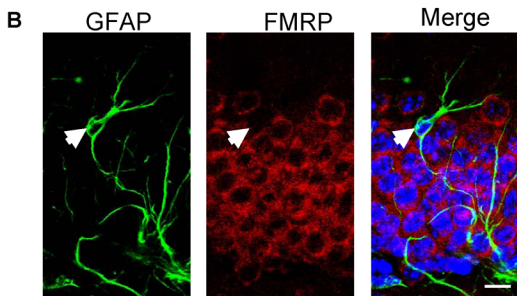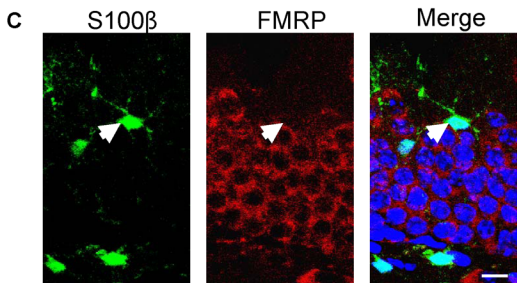

Supplement: Figure S1 — Fmrp is expressed in DG neurons but not astrocytes in the adult hippocampus. (A) Fmrp staining is prominent in the majority of the DG cells of WT mice but is absent in the KO mice. (B,C) Fmrp expression was nearly undetectable in GFAP (B) or S100β (C) expressing astrocytes. Arrows point to astrocyte that are negative for Fmrp staining. Scale bars = 10 µm. (1.47 MB PDF) [file pgen.1000898.s001.pdf]

**A**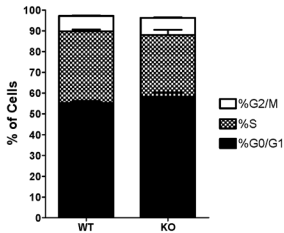**B****G0/G1 Phase**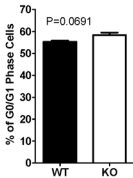**C****S Phase**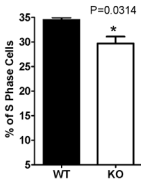**D****G2/M Phase**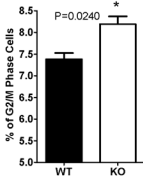

Supplement: Figure S2 — Adult brain-derived aNPCs from Fmr1 KO mice exhibited altered proliferation. (A) Single plain Laser Scanning Confocal image showing that adult brain-derived aNPCs cultured under proliferating conditions expressed neural progenitor markers: Nestin (cytoplasmic, red) and Sox2 (nuclear, green). Dapi was used to label nuclear DNA (blue). (B–E) Cell cycle profile of WT and Fmr1 KO aNPCs indicating that Fmr1 KO aNPCs had more cells in mitosis (G2/M phase) and fewer cells in S phase. N = 3 independent cell preparations. *, p<0.05, Student's t-test. Data is shown as mean ± SEM. (0.09 MB PDF) [file pgen.1000898.s002.pdf]

**A***Fmr1* mRNA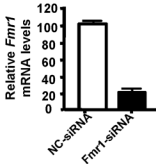**B**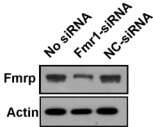

Supplement: Figure S3 — Fmr1-siRNA could specifically reduce the mRNA and protein expression of Fmrp as shown by real-time PCR (A) and Western blotting (B). (0.04 MB PDF) [file pgen.1000898.s003.pdf]

# aNPCs derived from adult DG

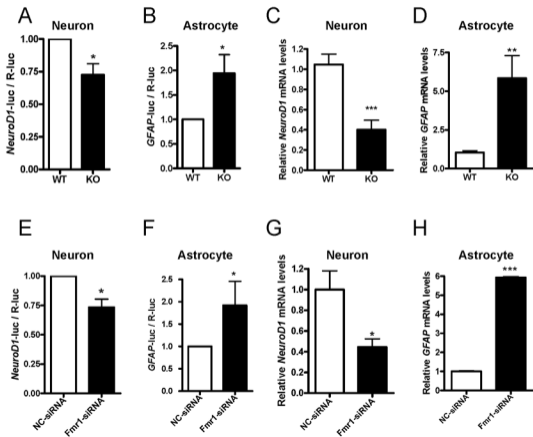

Supplement: Figure S4 — aNPCs isolated from the DG of Fmr1 KO mice had similar phenotypes as those found in aNPCs isolated from the Fm1 KO forebrain. (A,B) Fmr1 KO DG aNPCs exhibited lower NeuroD1 promoter (A) but higher GFAP promoter (B) activities. (C,D) Fmr1 KO DG aNPCs had lower levels of endogenous NeuroD1 mRNA (C) but higher levels of endogenous GFAP mRNA (D). (E–H) Acute knockdown of Fmrp expression in WT DG aNPCs using siRNA led to decreased neuronal promoter activity (E; mean ± SEM n = 6, p<0.05) and decreased NeuroD1 mRNA levels (F), but increased GFAP promoter activity (G; mean ± SEM n = 6, p<0.05) and increased GFAP mRNA levels (H; p<0.001). Therefore, Fmrp has similar functions in DG aNPCs compared to aNPCs derived from the forebrain. All data are shown as mean ± SEM. Statistics was done using two tailed unpaired Student's t-test. *, p<0.05; **, p<0.01; ***, p<0.001. NC-siRNA, nonsilencing control siRNA. (0.10 MB PDF) [file pgen.1000898.s004.pdf]

A

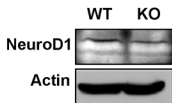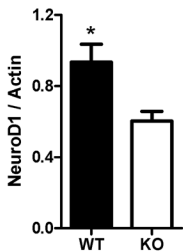

B

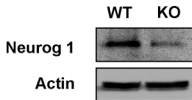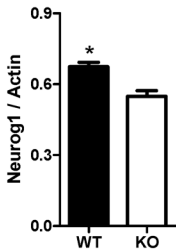

C

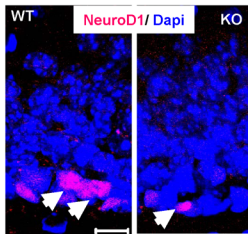

Supplement: Figure S5 — Reduced expression of NeuroD1 and Neurogenin1 in Fmr1 KO mice (A,B). The protein levels of two transcription factors specific to young neurons, NeuroD1 (A) and Neurog1 (B), exhibited lowered expression levels in Fmr1 KO hippocampus, as assessed by Western blot analysis. Sample images of Western blots are shown in the upper panels and quantification of 3 blots are shown in the lower panels. β-actin was used as a loading control. (C) Immuno histological staining using shows reduced number of NeuroD1-positive Cells (white arrows) in the subgranular zone of the DG. All data are shown as mean ± SEM. Statistics were done using two tailed unpaired Student's t-test. *,p<0.05; Scale bar = 10 µm. (0.38 MB PDF) [file pgen.1000898.s005.pdf]

## A Quantification of Western Blot

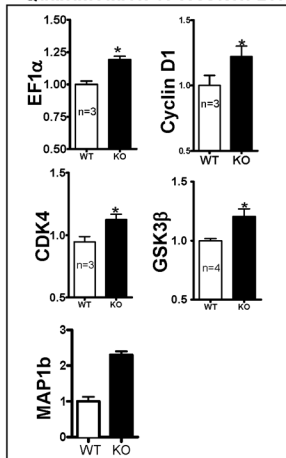

## B Real time PCR of mRNA

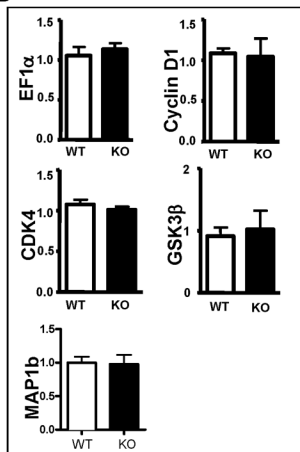

## C

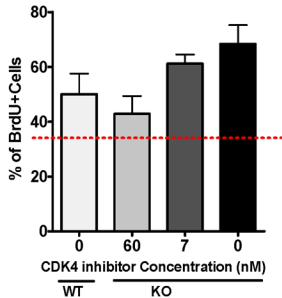

Supplement: Figure S6 — Expression analysis of proliferating Fmr1 KO aNPCs. (A) Quantification of Western blot band intensities (as shown in Figure 4C) normalized to ß-actin levels demonstrates increased protein levels of EF1a, CyclinD1, CDK4, GSK3β, and MAP1b in Fmr1 KO aNPCs. Data is from n = 3 or 4 independent measurements with KO levels normalized to the WT levels. Student's t-test was performed on data before normalization to ensure accurate statistical analysis. (B) The mRNA levels of EF1a, CyclinD1, CDK4, GSK3β, and MAP1b were not changed in proliferating Fmr1 KO aNPCs. The steady-state mRNA level determined by real-time PCR was normalized to18S. (C) CDK4 inhibitor was dissolved in DMSO (0 concentration). At 60 nM, this inhibitor can reverse the proliferation of Fmr1 KO aNPCs and bring it to the level of WT cells (n = 3), suggesting that increased CDK4 activity might be a reason for increased proliferation of Fmr1 KO aNPCs. Proliferation was assessed by BrdU pulse labeling followed by immunostaining and stereological quantification. All data are shown as mean ± SEM. Statistics were done using two tailed unpaired Student's t-test. *, p<0.05. (0.14 MB PDF) [file pgen.1000898.s006.pdf]

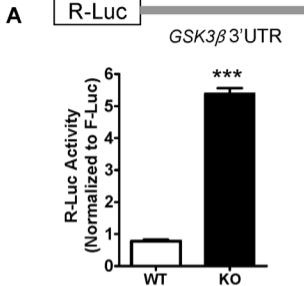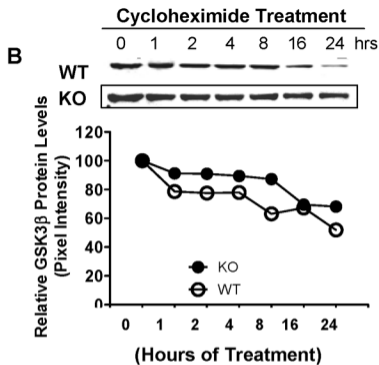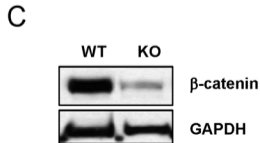

Supplement: Figure S7 — Fmrp regulates translation of GSK3β. (A) A GSK3β 3′untranslated region (3′UTR) was cloned into a Renilla luciferase (R-luc) expression vector (top panel) therefore the translation of R-luc was regulated by the 3′UTR of GSK3β. Transfection of this construct into aNPCs resulted in higher R-Luc activity (normalized to firefly luciferase internal control) in Fmr1 KO compared with WT cells (Data is shown as mean ± SEM; n = 3, p<0.001, Student's t-test), suggesting that elevated translational activity is directed by GSK3β 3′UTR in the absence of Fmrp. Data is shown as mean ± SEM. Statistics were done using two tailed unpaired Student's t-test. ***, p<0.001. (B) aNPCs were treated with a protein synthesis inhibitor, cycloheximide, during a 24 hour period. Gsk3β protein levels were determined using Western blot (top panel) and quantified. The result indicates that the degradation rate of GSK3β protein is not significantly different between Fmr1 KO and WT aNPCs. (C) β-catenin protein expression was decreased in proliferating Fmr1 KO aNPCs. PDF (35KB) (0.13 MB PDF) [file pgen.1000898.s007.pdf]

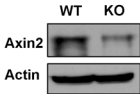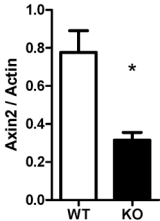

Supplement: Figure S8 — Reduced expression of Axin2 protein in the hippocampus of Fmr1 KO mice The protein levels of Axin2, a downstream effecter of canonical Wnt signaling pathway, exhibited lowered expression levels in Fmr1 KO hippocampus. Sample images of Western blots (left) and quantification of 3 blots (right) are shown. β-actin was used as a loading control. Data is shown as mean ± SEM. Statistics were done using two tailed unpaired Student's t-test. *, p<0.05. (0.04 MB PDF) [file pgen.1000898.s008.pdf]

A

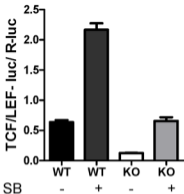

B

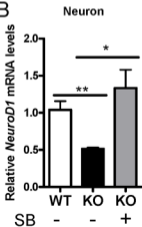

C

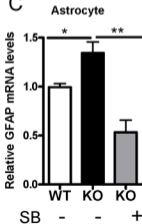

Supplement: Figure S9 — Gsk3β inhibitor could rescue the neuronal and astrocyte differentiation deficits of Fmr1 KO DG aNPCs. (A) Gsk3β inhibitor SB216763 (SB) SB could enhance the Wnt signaling in both WT and Fmr1 KO aNPCs. (B,C) SB could rescue the reduced NeuroD1 (A) mRNA levels and increased GFAP mRNA levels (B) in Fmr1 KO aNPCs. SB (dissolved in DMSO) was added at initiation of differentiation at 4 µM. Equal amount of DMSO was added to WT and KO control aNPCs. All data are shown as mean ± SEM. Statistics were done using two tailed unpaired Student's t-test. *, p<0.05; **, p<0.01; ***, p<0.001. (0.05 MB PDF) [file pgen.1000898.s009.pdf]

A

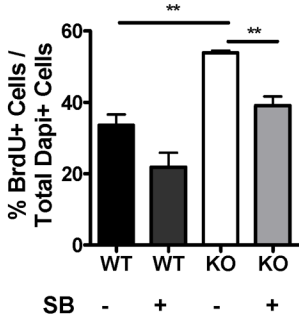

B

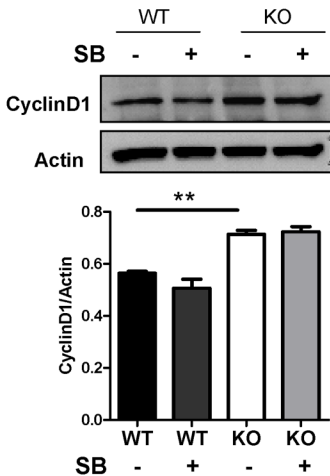

Supplement: Figure S10 — Gsk3β inhibitor could reverse the proliferation deficit of Fmr1 KO aNPCs. (A) Gsk3β inhibitor SB216763 (SB) SB could repress proliferation of Fmr1 KO aNPCs. Effect on WT cells was not statistically significant (p = 0.08). (B) SB treatment did not affect cyclin D1 expression levels in either WT or KO aNPCs (n = 3). All data are shown as mean ± SEM. Statistics were done using two tailed unpaired Student's t-test. *, p<0.05; **, p<0.01. PDF (53KB) (0.13 MB PDF) [file pgen.1000898.s010.pdf]

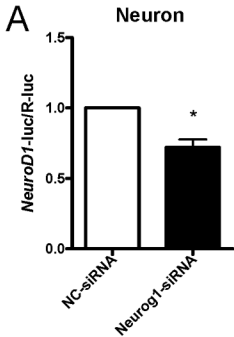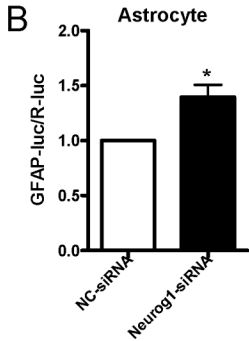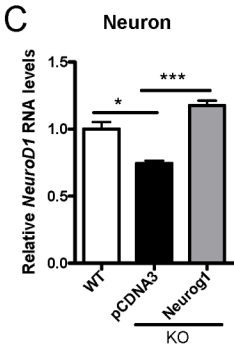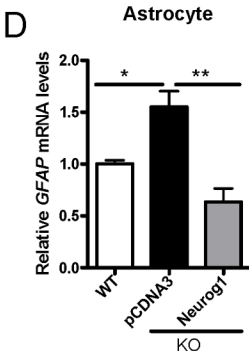

Supplement: Figure S11 — Neurog1 regulates the fate specification of the DG aNPCs. Neurog1 could rescue the neuronal (A) and astrocyte (B) differentiation deficits of Fmr1 KO DG aNPCs. Acute knockdown of Neurog1 in WT DG aNPCs led to reduced neuronal (C) but increased astrocyte (E) differentiation. NeuroD1 is an neuronal lineage marker. GFAP is an astrocyte lineage marker. The relative mRNA levels were in comparison with GAPDH mRNA. Promoter activities of NeuroD1 and GFAP (fire fly luciferase, luc) were normalized to a cotransfected internal control (E1a-Renilla luciferase, Rluc). All data are shown as mean ± SEM. Statistics were done using two tailed unpaired Student's t-test. *, p<0.05; **, p<0.01; ***, p<0.001. (0.10 MB PDF) [file pgen.1000898.s011.pdf]
