# Supplementary material for: Fragile X Mental Retardation Protein Regulates Proliferation and Differentiation of Adult Neural Stem/Progenitor Cells
Source: PLoS Genet. 2010 Apr 8;6(4):e1000898. doi: 10.1371/journal.pgen.1000898 (PMC2851565; doi:10.1371/journal.pgen.1000898)
Supplement: Text S1 — Supplemental methods. (0.13 MB PDF) [file pgen.1000898.s012.pdf]

Supplemental Methods

Isolation and cultivation of adult NPCs. aNPCs used in this study were isolated from 8- to 10-week-old male *Fmr1* KO mice and wild-type (WT) controls based on published methods: for forebrain aNPCs [1] and for DG aNPCs [2]. For forebrain cells, animals were sacrificed and the forebrains with olfactory bulb removed were dissociated mechanically. For DG cells, DG were dissected from 500 um coronal sections as described. After enzymatic digestion using MACS Neural Tissue Dissociation kit (Miltenyi Biotech, Germany), we added 5 ml of DMEM/F-12 containing 10% FBS (Sigma-Aldrich, #F 4135), 2 mM L-glutamine (GIBCO, #25030-081), and 1% Antibiotic-Antimycotic (GIBCO, #15240-062) into each sample to stop digestion. After filtering through a 70-µm cell strainer (BD Falcon, #252350, CA) and washing with DMEM/F-12 (2 mM L-Glutamine, 1% Antibiotic-Antimycotic), the single-cell suspension from each sample was loaded onto 50% Percoll. The NPCs were separated from other cells by ultracentrifugation at 127 krpm for 30 min at 20°C using a SW41 rotor (Beckman, CA). The fraction containing NPCs was collected and cultured with DMEM/F-12 medium containing 20 ng/ml basic fibroblast growth factor (FGF-2, PeproTech, #K1606), 20 ng/ml epidermal growth factor (EGF, PeproTech, #A2306), 1% N2 supplement (GIBCO, #17502-048), 1% Antibiotic-Antimycotic, and 2 mM L-glutamine in a 5% CO_2 incubator at 37°C. Half of the medium was replaced every two days.

Proliferation and differentiation analyses of cultured aNPCs.

Proliferation and differentiation of aNPCs were carried out using our established method[3,4]. We used only early passage cells (between passage 4 and 10) and only the

same passage numbers of WT and KO cells. For each experiment, triplicate wells of cells were analyzed, and results were averaged as one data point (n = 1). At least 3 independent experiments (n = 3) were performed and used for statistical analyses for each analysis. To study cell proliferation, we dissociated neural stem cells with trypsin and plated them on poly-L-ornithin/laminin-coated chamber slides (Nunc, #154526) at a density of 50,000 cells/well in proliferation medium (see above). At 20 h post-plating, 5 μM 5-bromo-2'-deoxyuridine (BrdU, Sigma-Aldrich) was added into the culture medium for 8 h. NPCs were then washed with PBS and fixed with 4% paraformaldehyde for 30 min at room temperature, followed by immunohistochemical analysis. To detect BrdU incorporation, fixed cells were pretreated with 1M HCl for 30 min at 37°C, and then washed with borate buffer, pH 8.5, for 30 min. We then followed our standard immunohistochemistry protocol[4].

For the differentiation assay, at 24 h post-plating, cells were changed into differentiation medium, DMEM/F12 (1:1), containing 5 μM forskolin (Fsk, Sigma-Aldrich, #F-6886), 1 μM retinoic acid (RA, Sigma-Aldrich, #R-2625), and sometimes with 0.5% fetal bovine serum (FBS, Sigma-Aldrich, #F-2442) for 4 days, followed by fixation with 4% paraformaldehyde for 30 min, then washing with Dulbecco's Phosphate-Buffered Saline, pH 7.4 (DPBS) for 30 min.

Immunocytochemistry staining was carried out as described[3,4]. Briefly, cells were preblocked using DPBS containing 5% normal goat serum (VECTOR, #S-1000) and 0.1% Triton X-100 for 30 min, followed by overnight incubation with primary antibodies: mouse neuron-specific type ß-III tubulin (Tuj1, 1:4000, Promega, #G712A), rabbit glial fibrillary acidic protein (GFAP, 1:1000, DAKO, #Z-0334), or rat anti-BrdU

(1:3000, Abcam, ab-6326). After washing with DPBS, cells were incubated with secondary antibodies that included goat anti-mouse Alexa Fluor 568 (1:500, Invitrogen, #A11031), goat anti-rabbit Alexa Fluor 647 (1:500, Invitrogen, #A21245), or goat anti-rat Alexa Fluor 568 (1:500, Invitrogen, #A11077), followed by counterstaining with the fluorescent nuclear dye 4',6-dimidino-2'-phenylindole dihydrochloride (DAPI, Sigma-Aldrich, #B2261). After the cells were mounted with VECTASHIELD (VECTOR, #H-1000), the numbers of Tuj1-, GFAP-, BrdU, or activated caspase-3-positive cells were quantified using an Olympus BX51 microscope equipped with a MicroFire digital camera (Optronics) and a motorized stage using a 20X objective lens. The quantification was carried out using an unbiased stereology method with assistance from StereoInvestigator software (MBF Biosciences). The percentage of differentiated cells was calculated as the number of Tuj1- or GFAP-labeled cells divided by the total number of cells stained with DAPI. The percentage of proliferating cells was defined as the number of BrdU-labeled cells divided by total DAPI-positive cells.

Cell cycle analysis. Cultured aNPCs were trypsinized and 10^6 cell were resuspended in 5ml PBS, followed by centrifugation for 5 min at 1000rpm. Cells were then resuspended in 0.5ml PBS fixed with 70% ethanol for more than 2 hour on ice. The fixed cells were then centrifuged, washed, and suspended in 1ml Propidium Iodide/Triton X-100 staining solution (0.1% Triton X-100, 0.2mg/ml DNase-free RNase A and 20ug/ml PI) and incubated at 37°C for 15 min. The cell cycle profile of labeled cells was determined by a Becton Dickinson flow cytometer

In vivo neurogenesis studies. In vivo neurogenesis analyses were performed essentially as described previously[1,4]. These experiments have been performed using 3

different batches of animals with n = 4-6/genotype each batch. For the first two batches, BrdU (50mg/kg) was injected to 8-week-old mice daily for 7 consecutive days to increase the amount of labeling. Mice were then euthanized 1 day post-injection to assess the in vivo proliferation (and early survival) of labeled cells. For cell survival analysis, another group of mice was injected with BrdU at 8 weeks of age and euthanized 4 weeks post-injection. The third batch of mice, on the other hand, were given 6 injections of BrdU (50mg/kg) within 24 hours to label all dividing cells in the DG within this time period and sacrificed at 4 hours post-last injection based on published protocol [5]. Mice were euthanized by intraperitoneal injection of sodium pentobarbital and then transcardially perfused with saline followed by 4% PFA. Brains were dissected out, post-fixed overnight in 4% PFA, and then equilibrated in 30% sucrose. Forty-μm brain sections were generated using a sliding microtone and stored in a -20°C freezer as floating sections in 96-well plates filled with cryoprotectant solution (glycerol, ethylene glycol, and 0.1M phosphate buffer, pH 7.4, 1:1:2 by volume).

We performed immunohistological analysis on 1-in-6 serial floating brain sections (240 μm apart) based on the published method[1]. The primary antibodies used were: rat-anti-BrdU (1:3000, Abcam, ab-6326), mouse anti-NeuN (1:5000; Chemicon International, Temecula, CA), rabbit anti-S-100β (1:500; Sigma), Ki67 (VECTOR, VP-K452), chicken anti-Nestin (1:1000, Aves, #mNES), and rabbit anti-GFAP (1:1000, Dako, Z0334), mouse anti-Fmrp (1:1000, Millipore clone 1C3), Sox2 (1:500, Santa Cruz), Doublecortin (1:200, Santa Cruz), NeuroD1(1:200, Santa Cruz). Fluorescent secondary antibodies were used at 1:250 dilutions (donkey from Jackson ImmunoResearch or goat from Invitrogen). After staining, sections were mounted,

coverslipped, and maintained at 4°C in the dark until analysis. BrdU-positive cells in the granule layer were counted using unbiased stereology (StereoInvestigator, MBF Biosciences, Inc) with a 5-μm guard zone, and the total number of BrdU+ cells in the DG was then calculated by StereoInvestigator, as described elsewhere [1,4]. The results were presented as number of BrdU+ cells in a cubic millimeter of dentate gyrus (DG). Ki67-positive cells in the subventricular zone (SVZ) were counted using unbiased stereology assisted by StereoInvestigator software (MBF Biosciences) as described[6]. Phenotype analysis of BrdU^{+} cells was performed as described previously[1]. Briefly, 50 BrdU^{+} cells in the DG were randomly selected, and their phenotypes (double labeling with either NeuN, S100β, GFAF, or Nestin) were determined using either a Zeiss LSM510 laser scanning confocal microscope or a Nikon TE2000 microscope equipped with a spin disc confocal microscope and MetaMorph quantification software. The data were analyzed using a Student's t-test (GraphPad software, www.graphpad.com).

DG volume was measured as described [7,8]. Briefly, one-in-six series of adjacent sections was stained with DAPI (4'-6-diaminodino-2-phenylindole, 1 μl/10 ml Tris-buffered saline for 10 min) to visualize the nuclei and to measure granule cell layer volume. The granule cell area was traced using a semiautomatic stereology system (StereoInvestigator, MicroBrightfield, Williston, VT) and a 20X objective (Olympus BX51). The DG volume was determined by the StereoInvestigator software by summing the traced granule cell areas for each section multiplied by the distance between sections sampled.

RNA immunoprecipitation (IP). RNA-IP was carried out as described [9]. Briefly, cultured aNPCs (2 X 10^6) were harvested and homogenized in 2 ml of ice-cold lysis buffer (10 mM Hepes [pH 7.4], 200 mM NaCl, 30 mM EDTA, and 0.5% Triton X-100) with 2X complete protease inhibitors (Boehringer-Mannheim) and 400 U/ml rRNasin (Promega). Nuclei and debris were pelleted at 3,000 X g for 10 min; the supernatant was collected and raised to 300 mM NaCl, and clarified at 14,000 X g for 30 min. The resulting supernatant was pre-cleared for 1 h with 100 µl recombinant protein G agarose (Invitrogen) (washed with lysis buffer first). An aliquot of precleared input was saved for RNA extraction (200 µl) and protein analysis (100 µl). A monoclonal antibody against Fmrp (7G1-1)[9] was incubated with recombinant protein G agarose at 4ºC for 2 h and washed 3 times with lysis buffer. RNase Inhibitors (Invitrogen) will be added to the remaining lysates. The precleared lysates were immunoprecipitated with antibody-coated recombinant protein G agarose at 4ºC overnight. After third wash with the lyses buffer, 10% of immunoprecipitate was saved for protein analysis. The remaining was washed one more time and the immunoprecipitate was re-suspended into Trizol (Invitrogen) for RNA isolation.

Microarray assay. RNA from precleared input lysate (Input) and immunoprecipitate (IP) was isolated with Trizol reagent (Invitrogen) and the RNeasy Mini Kit (QIAGEN). In addition, total RNA from cultured mice adult neural stem cells treated with or without actinomycin D was isolated with Trizol reagent (Invitrogen) and the RNeasy Mini Kit (QIAGEN). The cRNA "targets" were generated following instructions from Affymetrix (www.affymetrix.com). In immunoprecipitation experiments, one fifth of the precleared

input was used to generate input cRNA, and sixty percent of the IP RNA was used to generate IP-cRNA targets. These cRNAs were sequentially hybridized to the Rat Genome 230 2.0 array. In the actinomycin D experiments, cRNAs were hybridized to the Mouse 430_2 array. All the arrays were scaled to an average intensity of 500 and analyzed independently using GeneChip Operating Software (GCOS, Affymetrix). The mRNAs specifically associated with Fmrp in aNPCs were identified as described previously [9]. mRNAs enriched more than twofold in WT-IP over both WT-Input and *Fmr1* KO-IP were selected for further analyses.

Chemical treatment. Cycloheximide (Sigma) treatment was carried out as described[10]. Briefly, at 24 hours post plating, cycloheximide was added to culture medium at 50um. Cells were collected at different time points (from 1 hour to 24 hours). The cells were then lysed and 40 μg of proteins were loaded to each lane for Western blotting analysis using an antibody against Gsk3ß (see below). CDK4 inhibitor treatment was carried out as described (Li 2007, Zhu 2003). Briefly, CDK4 inhibitor (Calbiochem #219476) was added to proliferating aNPCs at 6 h post-plating. Cells were then pulse-labeled 16 hours later with BrdU for 8 hours in the presence of inhibitor, followed by fixation, staining, and stereological quantification. Gsk3ß inhibitor treatment was carried out as described [11]. Briefly, Gsk3ß inhibitor (SB216763, Sigma-Aldrich, dissolved in DMSO) was added to aNPCs upon initiation of differentiation with RA/Fsk (for NeuroD1 and Tuj1 assays) or RA/Fsk/FBS (for GFAP assay) *at* 4 μM final concentration. Cells were collected 2 days later for real time PCR analysis.

Real-time PCR. The first-strand cDNA was generated by reverse transcription with oligo dT primer or random hexamers. To quantify the mRNA levels with the real-time

PCR, aliquots of first-stranded cDNA were amplified with gene-specific primers and Power SYBR Green PCR Master Mix (Applied Biosystems) using a 7500 Fast Real-Time PCR System (Applied Biosystems). The PCR reactions contained 20-40 ng of cDNA (except the cDNA for the IP, for which 5% of the cDNA was used for each gene examined), Universal Master Mix (Applied Biosystems), and 300 nM of forward and reverse primers in a final reaction volume of 20 µl. The ratio of different samples was calculated by the data analysis software built in with the 7500 Fast Real-Time PCR System. The sequences of primer used are as the following:

Cyclin D1: forward: GCATGTTCGTGGCCTCTAAG; reverse: GTAGATGCACAACTTCTCGGC

CDK4: forward: CCGTCAGCACAGTTCGTGAG; reverse: CCATCAGCCGTACAACATTGG

EF1a: forward: CACCGAGCCACCATACAGTC; reverse: CTTAATGTAGGTGCTGACTTCC

Map1b: forward: GATTACAACGCTTCCGCCTC; reverse: GTCTTCTTCCATGGACGAAGG

Gsk3β: forward: GGGACAGTGGTGTGGATCAG; reverse: CTTGTTGGTGTTCCTAGGAC

NeuroD1: forward: TTAAATTAAGGCGCATGAAGGCC; reverse: GGACTGGTAGGAGTAGGGATG

Tuj1: forward: TATGAAGATGATGACGAGGAATCG; reverse: TACAGAGGTGGCTAAAATGGGG

GFAP: forward: CCAAGCCAAACACGAAGCTAA; reverse: CATTTGCCGCTCTAGGGACTC

Aquaporin: forward: CTTTCTGGAAGGCAGTCTCAG; reverse: CCACACCGAGCAAAACAAAGAT

References for Supplemental Information

1. Zhao X, Ueba T, Christie BR, Barkho B, McConnell MJ, Nakashima K, Lein ES, Eadie BD, Willhoite AR, Muotri AR, Summers RG, Chun J, Lee KF, Gage FH (2003) Mice lacking methyl-CpG binding protein 1 have deficits in adult neurogenesis and hippocampal function. Proc Natl Acad Sci U S A 100: 6777-6782.
2. Babu H, Cheung G, Kettenmann H, Palmer TD, Kempermann G (2007) Enriched monolayer precursor cell cultures from micro-dissected adult mouse dentate gyrus yield functional granule cell-like neurons. PLoS ONE 2: e388.
3. Barkho BZ, Song H, Aimone JB, Smrt RD, Kuwabara T, Nakashima K, Gage FH, Zhao X (2006) Identification of astrocyte-expressed factors that modulate neural stem/progenitor cell differentiation. Stem Cells Dev 15: 407-421.
4. Smrt RD, Eaves-Egenes J, Barkho BZ, Santistevan NJ, Zhao C, Aimone JB, Gage FH, Zhao X (2007) Mecp2 deficiency leads to delayed maturation and altered gene expression in hippocampal neurons. Neurobiol Dis 27: 77-89.
5. Hayes NL, Nowakowski RS (2002) Dynamics of cell proliferation in the adult dentate gyrus of two inbred strains of mice. Brain Res Dev Brain Res 134: 77-85.
6. Thored P, Wood J, Arvidsson A, Cammenga J, Kokaia Z, Lindvall O (2007) Long-term neuroblast migration along blood vessels in an area with transient angiogenesis and increased vascularization after stroke. Stroke 38: 3032-3039.
7. Kempermann G, Brandon EP, Gage FH (1998) Environmental stimulation of 129/SvJ mice causes increased cell proliferation and neurogenesis in the adult dentate gyrus. Curr Biol 8: 939-942.
8. van Praag H, Schinder AF, Christie BR, Toni N, Palmer TD, Gage FH (2002) Functional neurogenesis in the adult hippocampus. Nature 415: 1030-1034.
9. Brown V, Jin P, Ceman S, Darnell JC, O'Donnell WT, Tenenbaum SA, Jin X, Feng Y, Wilkinson KD, Keene JD, Darnell RB, Warren ST (2001) Microarray identification of FMRP-associated brain mRNAs and altered mRNA translational profiles in fragile X syndrome. Cell 107: 477-487.
10. Hongisto V, Vainio JC, Thompson R, Courtney MJ, Coffey ET (2008) The Wnt pool of glycogen synthase kinase 3beta is critical for trophic-deprivation-induced neuronal death. Mol Cell Biol 28: 1515-1527.
11. Coghlan MP, Culbert AA, Cross DA, Corcoran SL, Yates JW, Pearce NJ, Rausch OL, Murphy GJ, Carter PS, Roxbee Cox L, Mills D, Brown MJ, Haigh D, Ward RW, Smith DG, Murray KJ, Reith AD, Holder JC (2000) Selective small molecule inhibitors of glycogen synthase kinase-3 modulate glycogen metabolism and gene transcription. Chem Biol 7: 793-803.
